# Global analysis of expression, maturation and subcellular localization of mouse liver transcriptome identifies novel sex-biased and TCPOBOP-responsive long non-coding RNAs

**DOI:** 10.1186/s12864-021-07478-5

**Published:** 2021-03-24

**Authors:** Christine N. Goldfarb, David J. Waxman

**Affiliations:** Department of Biology and Bioinformatics Program, Boston University, 5 Cummington Mall, Boston, MA 02215 USA

**Keywords:** lncRNAs, TCPOBOP, Xenobiotic exposure, Sex-bias, Transcript maturation, Cellular fractionation, Nuclear fractionation, Chromatin-bound RNA

## Abstract

**Background:**

While nuclear transcription and RNA processing and localization are well established for protein coding genes (PCGs), these processes are poorly understood for long non-coding (lnc)RNAs. Here, we characterize global patterns of transcript expression, maturation and localization for mouse liver RNA, including more than 15,000 lncRNAs. PolyA-selected liver RNA was isolated and sequenced from four subcellular fractions (chromatin, nucleoplasm, total nucleus, and cytoplasm), and from the chromatin-bound fraction without polyA selection.

**Results:**

Transcript processing, determined from normalized intronic to exonic sequence read density ratios, progressively increased for PCG transcripts in going from the chromatin-bound fraction to the nucleoplasm and then on to the cytoplasm. Transcript maturation was similar for lncRNAs in the chromatin fraction, but was significantly lower in the nucleoplasm and cytoplasm. LncRNA transcripts were 11-fold more likely to be significantly enriched in the nucleus than cytoplasm, and 100-fold more likely to be significantly chromatin-bound than nucleoplasmic. Sequencing chromatin-bound RNA greatly increased the sensitivity for detecting lowly expressed lncRNAs and enabled us to discover and localize hundreds of novel regulated liver lncRNAs, including lncRNAs showing sex-biased expression or responsiveness to TCPOBOP a xenobiotic agonist ligand of constitutive androstane receptor (Nr1i3).

**Conclusions:**

Integration of our findings with prior studies and lncRNA annotations identified candidate regulatory lncRNAs for a variety of hepatic functions based on gene co-localization within topologically associating domains or transcription divergent or antisense to PCGs associated with pathways linked to hepatic physiology and disease.

**Supplementary Information:**

The online version contains supplementary material available at 10.1186/s12864-021-07478-5.

## Background

Since the discovery of more than a thousand novel, poly-adenylated long non-coding RNAs (lncRNAs) in mouse and human cells [1], lncRNAs have increasingly been shown to play key roles in gene regulation and disease states, including liver disease [2–4]. LncRNAs typically have 5′ caps, are transcribed by RNA polymerase II, and have polyA tails, and the DNA from which they are transcribed can have promoter-like or enhancer-specific histone modifications [1, 5]. Many lncRNA genes display striking patterns of developmental regulation and tissue-specific expression, which enables them to serve as condition-specific regulators of diverse biological processes [6]. LncRNAs can regulate cellular functions at multiple levels, including epigenetic modification and chromatin remodeling, transcriptional regulation, alternative splicing and mRNA translation [7–9]. For example, the lncRNA Xist, which is crucial for X-chromosome dosage compensation in female cells in eutherian mammals, introduces a repressed chromatin state marked by extensive histone-H3 K27me3 across one of the X-chromosomes, leading to X-inactivation and Barr body formation [10, 11], while the oncogenic lncRNA HOTAIR promotes cancer metastasis in part by silencing HOXA genes by promoting K27-trimethylation and K4-demethylation of histone-H3 [12]. In the liver, lncRNAs have been linked to liver fibrosis through their effects on glucose metabolism (LincIRS2) [13] and hepatic stellate cell regulation (H19, Meg3, HOTTIP) [14–16]. However, the biological functions and mechanisms of action of the vast majority of lncRNAs expressed in liver and other tissues are unknown.

Many lncRNAs are preferentially localized in the nucleus, where they can be visualized by single molecule RNA fluorescence in situ hybridization (smFISH) [17]. A few dozen lncRNAs have thus been characterized and show diverse patterns of expression, ranging from one or two distinct nuclear foci per cell to many individual RNA molecules throughout the nucleus and/or cytoplasm [18]. Multiplex error-robust FISH enables a higher throughput visualization of lncRNA and mRNA transcripts, though probes still need to be designed individually for each RNA of interest [19, 20]. LncRNAs can also be localized by RNA-seq analysis of subcellular RNA fractions, which is most often analyzed to give relative lncRNA concentrations in each cell fraction [21]. In prior studies from this laboratory, poly-adenylated RNA was isolated from nuclei purified from fresh mouse liver and sequenced to identify liver-expressed lncRNAs [22, 23]. Other studies using rRNA-depleted RNA from human hepatocellular carcinoma cell lines showed nuclear enrichment of lncRNAs, but not of RNAs coding for protein-coding genes (PCGs) [24]. While a majority of well-studied lncRNAs appear to function primarily in the nucleus, there are many well described cytoplasmic lncRNAs with cytoplasmic functions, such as miRNA sponging and regulation of mRNA stability and translational efficiency; furthermore, even nuclear lncRNAs may be present in the cytoplasm at significant levels [21, 25]. Studies of lncRNA localization are a critical step for characterization and elucidation of cell compartment-dependent functions for newly discovered lncRNAs, including the thousands of novel lncRNAs that we recently identified in mouse and rat liver [22, 26, 27].

Within the nucleus, lncRNAs may be nucleoplasmic, may be associated with the nuclear matrix or other structures, or may be tightly bound to chromatin, where they can interact directly with chromatin modifying complexes and regulate transcription. LncRNAs that bind to specific chromatin modifying complexes have been identified by RNA immunoprecipitation, although there are concerns about promiscuity and non-specific binding [28]. Related technologies have enabled the discovery of the specific RNAs, proteins and genomic regions that interact with individual lncRNAs [29], but this approach is not readily applied on a global scale to study the thousands of lncRNAs expressed in a given cell line or tissue. However, by fractionating nuclei using a high urea buffer containing salts and detergent, RNAs that are tightly bound to chromatin can be separated from RNAs that are soluble in the nucleoplasm, enabling the characterization of several thousand lncRNAs enriched in the insoluble chromatin fraction, as implemented in human cell lines and mouse macrophages [30, 31].

We previously identified 15,558 lncRNAs expressed in mouse liver under a variety of biological conditions [22, 26]. Tissue-specific expression patterns, epigenetic states, and regulatory elements, including nearby regions of chromatin accessibility and liver-specific transcription factor binding were determined for a subset of these lncRNAs [23]. A few hundred liver-expressed lncRNA genes were shown to respond to pituitary growth hormone secretory patterns [23], a key factor regulating sex-biased gene expression in the liver [32–34]. Furthermore, sex and strain-dependent genetic regulation was characterized in livers of Diversity Outbred mice [35], where co-expression network analysis identified sex-biased lncRNAs likely to control sex-biased PCG expression through negative regulatory mechanisms [26]. Furthermore, liver lncRNAs that respond to xenobiotic exposure and may impact xenobiotic toxicity have been identified [22, 36–39] and were closely linked to xenobiotic dysregulation of pathways involving fatty acid metabolism, cell division and immune responses [27]. However, key information regarding subcellular localization is lacking for the vast majority of liver-expressed lncRNAs, which complicates efforts to determine whether they have regulatory or other cellular functions in the cytoplasm, nucleoplasm or when bound to chromatin.

Here, we use RNA-seq to characterize expression patterns for a set of 15,558 liver-expressed lncRNAs with known gene and isoforms structures [26] and compare them to those of some 20,000 PCGs across four subcellular fractions and under four different biological conditions. We identify lncRNAs, as well as PCGs, whose transcripts are present at significantly different levels/different relative concentrations between the cytoplasm and the nucleus, and for nuclear transcripts, between the nucleoplasm and a chromatin-bound fraction. We find a strong enrichment of thousands of liver-expressed lncRNAs in the chromatin fraction, including lncRNAs that respond to endogenous hormonal factors or external chemical exposures, many expressed at too low a level for discovery by traditional RNA-seq analysis of whole liver tissue or even in purified liver nuclei. Our analysis of these rich datasets gives new insights into the maturation of hepatic lncRNA transcripts, and integration of our findings with prior work enabled us to identify hormonally regulated as well as xenobiotic-responsive lncRNAs that are promising candidates for future investigations of lncRNA function in liver biology and disease.

## Results

### Gene expression analysis in liver subcellular fractions

**Fig. 1:**
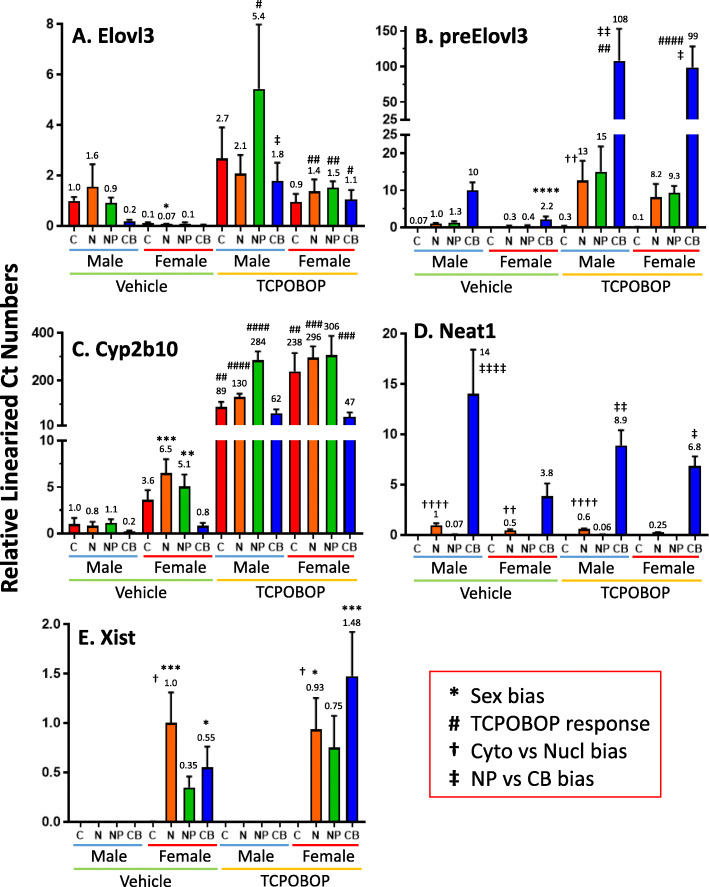
qPCR analysis of liver subcellular fractions using select marker genes. Expression of each gene was determined by qPCR across the cytoplasm (C), nucleus (N), nucleoplasm (NP) and chromatin-bound (CB) fractions. Data shown are relative expression levels (values above each bar, with one of the bar set = 1.0 for each gene, as marked), which are presented as mean values + SEM for *n* = 4 mice per biological condition: vehicle treated male and female mice, and TCPOBOP-treated male and female mice. **a** Elovl3: male-biased expression seen in vehicle control group mice is largely lost following TCPOBOP treatment. **b** PreElovl3, assayed using qPCR primers that span an intron/exon boundary to amplify unspliced transcripts, which were significantly enriched in the chromatin-bound fraction after TCPOBOP exposure in both sexes. **c** Cyp2b10, validating TCPOBOP induction response, and also female-biased expression in the basal state. **d** *Neat1 (lnc14746),* highly chromatin-bound, validates the separation of nucleoplasmic and chromatin fractions. **e** The female-specific Xist (*lnc15394)*, strong expression in all three nuclear-derived fractions. Significance was determined by one-way ANOVA with Bonferroni correction, for four separate analyses, which are specified using four different symbols (red box), as follows: *p* < 0.05, one symbol; *p* < 0.01, two symbols; *p* < 0.001, three symbols; and *p* < 0.0001, four symbols. qPCR primers are shown in Table S1A

We sought to identify liver-expressed genes whose transcripts are differentially enriched between the cytoplasmic and nuclear compartments. We analyzed frozen liver obtained from untreated male and female mice, and from mice exposed to TCPOBOP, a specific CAR agonist ligand [40] that induces or represses several hundred genes in liver [22, 41]. Liver tissue was homogenized under conditions expected to preserve nuclear membrane integrity, and cytoplasmic and nuclear RNA then purified from the cytoplasmic lysate and nuclear pellet, respectively. Nucleoplasmic RNA was extracted from the isolated nuclei with high salt buffer and urea, and the insoluble chromatin pellet was digested with DNase followed by Trizol extraction of the released chromatin-bound RNA (Figure S1). RNA from each subcellular fraction was analyzed by qPCR to determine the localization and regulated expression of select sex-biased and TCPOBOP-responsive marker genes (Fig. 1). In untreated liver, *Elovl3* showed strong, male-biased expression in the cytoplasmic, nuclear and nucleoplasmic fractions (Fig. 1a). TCPOBOP induced *Elovl3* expression in the chromatin-bound RNA fraction in male liver, and in all four fractions in female liver, which largely abolished its sex-dependent expression. The primary transcript, pre-*Elovl3* RNA was highest in the chromatin-bound fraction and was induced > 10-fold by TCPOBOP in all three nuclear-derived fractions, consistent with induction of *Elovl3* gene transcription (Fig. 1b). The differential enrichment of mature *Elovl3* vs. pre-*Elovl3* RNA in each subcellular fraction validates the separation of the fractions. Further validation was obtained by examining *Cyp2b10*, which showed female-biased expression in untreated liver and was strongly induced by TCPOBOP (up to 300-fold) in both sexes (Fig. 1c). The lncRNA *Neat1* (*lnc14746*) was exclusively found in the nuclear and chromatin-bound fractions (Fig. 1d). *Xist* (*lnc15394*), which is only expressed in female cells, was found at similar levels in the nuclear, nucleoplasmic and chromatin-bound fractions and was absent from cytoplasm (Fig. 1e), as expected [18]. Thus, the cytoplasmic fraction is not contaminated by nuclear RNAs released during nuclear membrane break down [21].

To obtain a global view of the localization and regulation of liver-expressed lncRNA genes, we prepared RNA-seq libraries from polyA-selected RNA from each of the four fractions. We also sequenced the chromatin-bound fraction without polyA-selection to obtain expression data for both poly-adenylated and non-poly-adenylated RNAs, including transcripts that did not yet undergo polyadenylation. In all, we sequenced 65 RNA-seq samples representing the 5 cellular fractions under 4 different biological conditions (male and female liver, with and without TCPOBOP exposure) (Table S1A). These datasets were then analyzed to address questions related to lncRNA maturation, localization and regulation, as described below.

### Transcript maturity in different subcellular fractions

We used the following approach to assess relative transcript maturity for each liver-expressed, multi-exonic lncRNA and PCG (Table S1D). Reads mapping to exonic features (exon collapsed regions, EC), and separately, reads mapping to intronic only (IO) regions, were counted for each gene, and then normalized by the % exonic and % intronic length of the gene, respectively. The resultant normalized exonic and intronic read densities were used to calculate an intronic to exonic read density ratio, IO/EC (Table S1F). For a transcript that is completely unspliced (i.e., a primary, immature transcript), RNA sequence reads will be spread equally across the entire gene length, and the IO/EC ratio will equal 1; and for a transcript that is fully spliced, the intronic read count, and hence the IO/EC ratio, will equal 0. Thus, lower IO/EC ratios are associated with an apparent increase in transcript processing (increased RNA maturity).

**Fig. 2:**
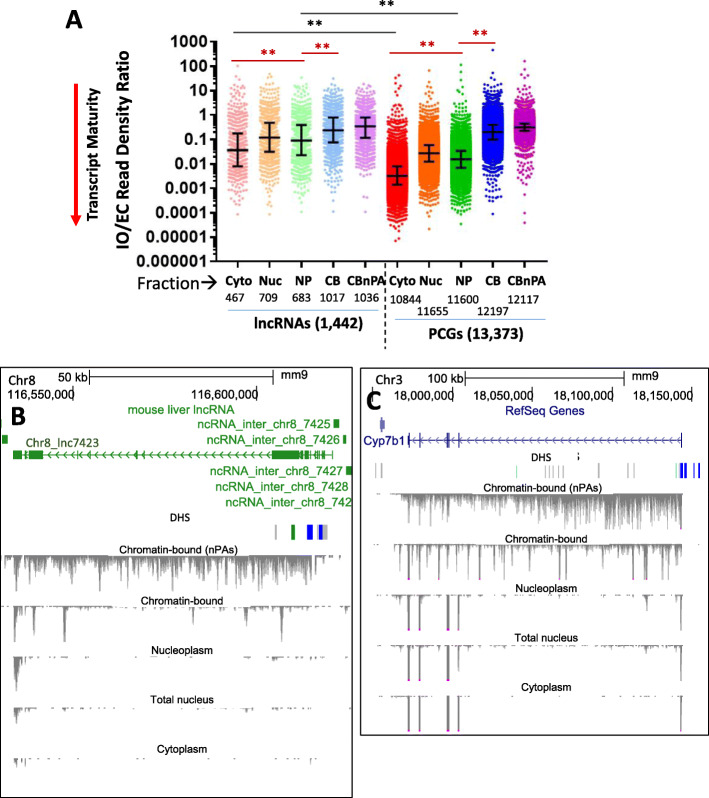
Transcript maturity across subcellular fractions determined by IO/EC read density ratio. **a** Distributions of IO/EC read density ratios for individual genes in vehicle-treated male liver, calculated from the weighted normalized read density values for IO and EC reads for each of 1442 multi-exonic lncRNAs (left) and 13,737 multi-exonic PCGs (right). IO/EC ratios displayed are mean values for *n* = 3 livers. The number of genes expressed in each subcellular fraction (see Methods) is listed below each column: cytoplasm (Cyto), nucleus (Nuc), nucleoplasm (NP), chromatin-bound (CB), chromatin bound non-PolyA selected (CBnPAs). Median IO/EC ratios (black horizontal midline) and IQR (error bars) are marked, and were lower for PCGs than lncRNAs: median cytoplasmic ratio = 0.0032 and 0.036, median nuclear ratio = 0.027 and 0.12, and median nucleoplasmic ratio = 0.015 and 0.089, for PCGs and lncRNAs, respectively (all significant at adjusted *p*-value < 0.0001). Median IO/EC did not differ between polyA-selected PCGs and lncRNAs (0.20 and 0.23, respectively) or between non-polyA-selected PCGs and lncRNAs (0.31 and 0.34, respectively). Black horizontal lines compare distributions of IO/EC ratios for lncRNAs vs PCGs in the cytoplasmic and nucleoplasmic fractions (other comparisons were not performed); red horizontal lines compare distributions between the indicated fractions for lncRNAs, and separately, for PCGs (** = adjusted *p*-value < 0.0001). The higher IO/EC ratios apparent for nuclear compared to nucleoplasmic transcripts is due to the nuclear fraction being a composite of both nucleoplasmic and chromatin-bound RNA. An excess of normalized intronic reads (IO/EC ratios > 1) is seen for a subset of genes, most notably chromatin-bound PCGs and all five lncRNA fractions. Many of these genes are lowly expressed (very low normalized EC reads), but have short, unannotated expressed features; others have intronic regions that overlap an exon of an expressed gene, leading to an artefactually high IO read count and hence IO/EC ratio. The data used to generate these graphs are found Table S1F. Figure S2 shows similar results for vehicle-treated female liver. **b** and **c** UCSC Browser screen shot showing BigWig files of minus strand sequence reads for each of the five indicated subcellular fractions for *lnc7423* (gene structure shown in green) and *Cyp7b1* in untreated male mouse liver. Extensive reads seen across the gene body in the chromatin bound fraction are substantially depleted after polyA-selection (top vs second reads track); however, multiple distinct peaks within intronic regions remain. BigWig Y-axis scale: 0 to − 25, except for non-polyA-selected track, which is 0 to − 5 (B) or 0 to − 12 (C). Both genes show male-biased expression, with many fewer sequence reads in corresponding fractions from female liver (not shown). Cytoplasm, and to a lesser extent nucleoplasm, are depleted of sequence reads for *lnc7423* but not for the PCG *Cyp7b1*, where a progressive increase in transcript maturity is apparent. These same patterns were seen in all three biological replicates. DHS, DNase hypersensitivity sites, indicating open chromatin. DHS showing significantly greater accessibility in male liver are marked in blue [42]. Figure S2 shows BigWig data for two female-specific genes

Median IO/EC ratios were highest in the chromatin-bound fractions and did not differ between PCGs and lncRNAs (Fig. 2a). Thus, the chromatin-bound fraction contains many more unspliced or partially spliced transcripts, in particular in the non-polyA selected fraction (Fig. 2b, c, Figure S2). IO/EC ratios decreased significantly in going from chromatin to the nucleoplasm, and from the nucleus to the cytoplasm, with the decreases being much greater for PCG than for lncRNA gene transcripts (Fig. 2a). Thus, liver transcripts are apparently the least spliced/most immature when bound to chromatin, and undergo a progressive increase in maturation as they transit through the nucleoplasm and on the cytoplasm. Furthermore, lncRNA splicing was apparently less efficient/less complete than PCG splicing, with median IO/EC ratios up to 11-fold higher for lncRNAs than for PCGs. This is consistent with reports that at least some incompletely spliced lncRNAs are biologically active (see Discussion).

### Differential enrichment of transcripts in cytoplasm vs nucleus

**Fig. 3:**
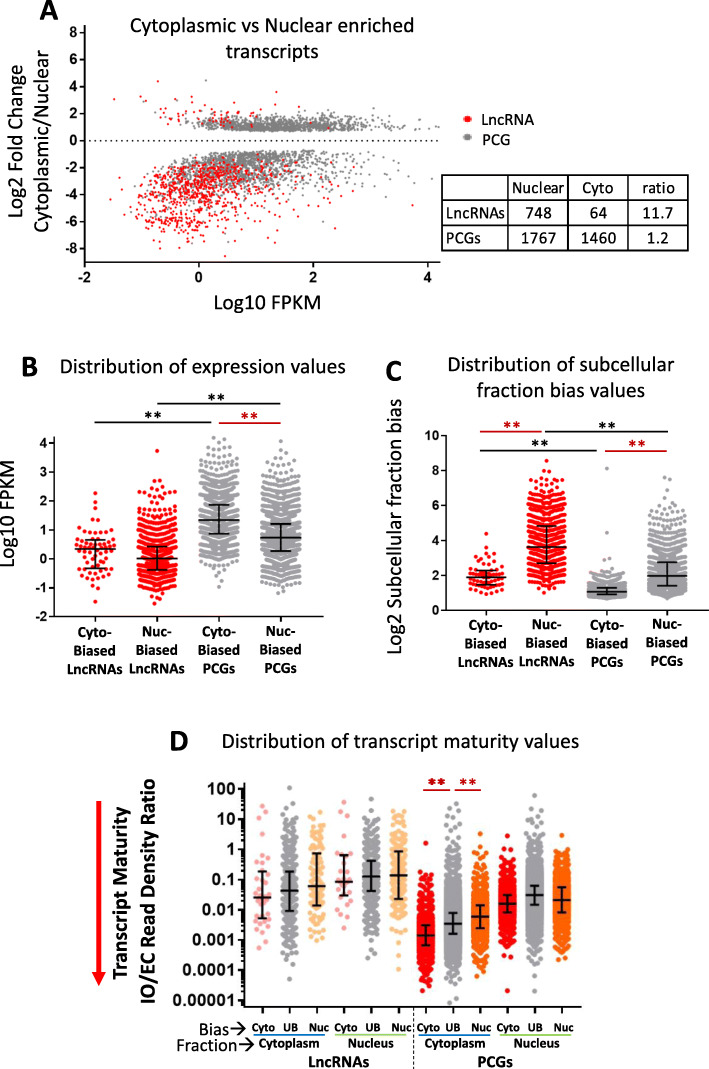
Expression, subcellular fraction enrichment and maturity of cytoplasmic versus nuclear transcripts. **a** Subcellular fraction enrichment (compartment bias) displayed as normalized cytoplasmic (Cyto) to nuclear (Nuc) expression ratio, of all RNAs that show either cytoplasmic-biased (positive y-axis) or nuclear-biased transcript levels (negative y-axis) at an edgeR-adjusted *p*-value < 0.001 in at least one of the four biological conditions assayed. For genes showing significant compartment bias in more than one biological condition, data is shown for the condition with the highest FPKM value (Table S2A, columns D and E). Each data point represents one gene showing nuclear or cytoplasmic bias (gene counts shown in table at right). Data are graphed separately for lncRNAs and PCGs in Figure S3A-S3B. **b** Distributions of FPKM values, and **c** distribution of subcellular fraction bias values (i.e., differential expression values) for the four indicated sets of subcellular fraction-enriched transcripts. The median fraction bias was 1.8–1.9-fold higher (adjusted p-value < 0.0001) for the nuclear-biased transcripts than for the cytoplasmic-biased transcripts. **d** Distributions of transcript maturity values (normalized IO/EC read density ratios, from Table S1F) in the cytoplasmic and nuclear fractions (“Fraction”) for multi-exonic lncRNAs and multi-exonic PCGs that show a significant cytoplasmic bias (Cyto) or nuclear bias (Nuc) (“Bias”), or that do not show a significant compartment bias (UB, unbiased). For **b**, **c**, and **d**, median values (black horizontal midline) and IQR (error bars) are indicated; black horizontal lines compare lncRNAs to PCGs within the same fraction, and red horizontal lines compare lncRNAs, or PCGs, between groups, as marked, with ** indicating adjusted *p*-value < 0.0001. In **d**, statistical analysis was used to compare Cyto vs UB, and UB vs Nuc, for lncRNAs and PCGs, based on expression data in the cytoplasm or in the nucleus

We sought to identify RNAs that showed differential intracellular localization, as indicated by significant differential expression between subcellular fractions. RNA-seq samples were normalized across samples based on total reads mapping to exons (EC read counts), and differential expression analysis was used to identify transcripts significantly enriched at high stringency (adjusted *p* < 0.001) in cytoplasmic vs nuclear fractions, and separately, in nucleoplasmic vs chromatin-bound fractions, and in the chromatin-bound fractions with vs without polyA selection (Tables S2A-S2C). We found many more lncRNA transcripts were significantly enriched in nuclear RNA (*n* = 748) than enriched in cytoplasmic RNA (*n* = 64) (11.7-fold difference vs. only 1.2-fold difference for PCGs; Fig. 3a, Figure S3AB). Overall, the median expression level was 51-fold lower for the nuclear-biased lncRNAs as compared to the nuclear-biased PCGs (Fig. 3b). Furthermore, much higher subcellular fraction expression ratios were found for the nuclear-biased transcripts than for the cytoplasmic-biased transcripts, most notably for the lncRNAs (Fig. 3c). The strong apparent nuclear enrichment of many lncRNAs (median nuclear to cytoplasmic ratio = 12.5-fold; IQR, 6.5 to 22.3) contrasts with a much weaker cytoplasmic bias for PCGs (median cytoplasmic to nuclear ratio = 2.1-fold; IQR, 1.89 to 2.45) (Fig. 3c), where ongoing nuclear transcription to generate a robust basal level of primary transcripts would effectively dampen the cytoplasmic to nuclear ratio.

Transcript maturity may in part drive these differences in expression between subcellular fractions, at least for PCGs. Thus, PCG transcripts enriched in the cytoplasm are on average more mature (lower median IO/EC ratio) than the corresponding fraction-unbiased and nuclear-enriched transcripts (Fig. 3d). This greater maturity of cytoplasm-enriched PCG transcripts is associated with a significantly shorter gene length, but not a lower percentage of intronic sequence (Figure S3C, Figure S3D). In the nucleus, transcript maturity was similar, or even higher, for nuclear-biased PCGs and lncRNAs as compared to non-compartment-biased PCGs and lncRNAs (Fig. 3d). This suggests that other factors, such as chromatin binding, examined below, contribute to transcript enrichment in the nucleus.

### Widespread enrichment of lncRNAs in chromatin-bound RNA

**Fig. 4:**
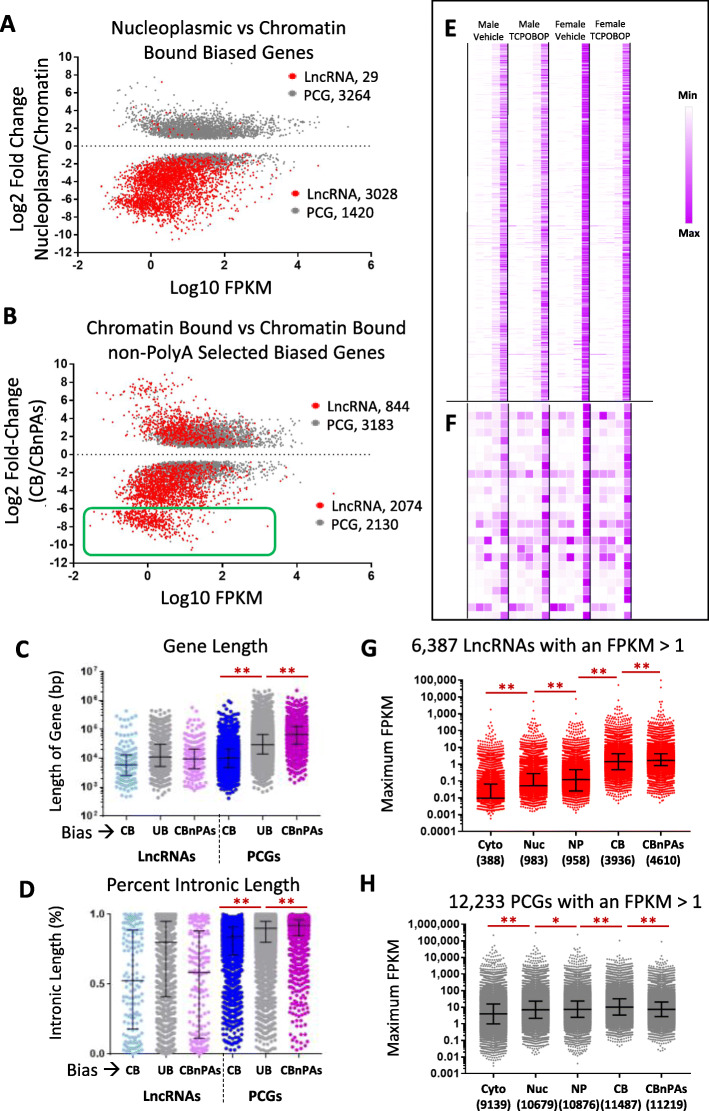
Differential expression of lncRNAs and PCGs across nuclear subcellular fractions. Subcellular fraction bias between: **a** nucleoplasm (NP) and the chromatin-bound (CB) fraction; or **b** within the chromatin-bound fraction, between polyA-selected and non-polyA selected RNA, based on an edgeR adjusted p-value < 0.001 in at least one of the four biological conditions assayed (Table S2B and Table S2C, columns D and E). Gray dots are PCGs, red dots are lncRNAs; numbers of genes whose transcripts are enriched in each fraction are shown above and below the dashed line, respectively. For any gene showing a significant bias in more than one biological condition, data is shown for the condition with the highest FPKM value. In **b**, green box encompasses CBnPAs-biased genes with log2 fold-change < − 6, which are further analyzed in **e** and in **f.  c** and **d**, Distributions of gene lengths (**c**) and percent intronic length (**d**) for chromatin-bound biased, non-compartment-biased (UB, unbiased) and CBnPAs-biased, graphed separately for lncRNAs and PCGs; also see Table S1D, columns M-Q. Significant differences for PCGs are as marked; no significant differences were seen for lncRNAs. See Figure S4 for corresponding data for NP-biased vs CB-biased genes, and Figure S5 for CB-biased genes, with vs without polyA selection. **e** and **f**, Normalized expression for the 506 lncRNAs (**e**) and 26 PCGs (**f**) that were very strongly CBnPAs-biased (genes from green box in **b**) across all 4 biological conditions (marked at top), for each of 5 subcellular fractions (columns from left to right within each condition: Cytoplasm, Nucleus, Nucleoplasm, Chromatin-bound, and Chromatin-bound non-PolyA-selected). See data in Table S2C, columns AD-AS. Data are shown for expression of each gene (row), normalized to the highest expression of that gene in a single condition and fraction. Seventeen of the 506 lncRNAs show sex-biased expression (Table S3A), and 19 show TCPOBOP-responsiveness (Table S3B) in at least one fraction. **g** and **h**, Distribution of expression values (FPKM) for the subsets of 6387 lncRNAs (**g**) and 12,233 PCGs (**h**) expressed at FPKM > 1 in at least one of the 5 subcellular fractions. The maximum expression of the gene across the four biological conditions is graphed for each subcellular fraction. Only a subset of the lncRNAs and PCGs were expressed at FPKM > 1 in each fraction (gene count numbers below each column). Based on expression data in Tables S2A-S2C. Median FPKM values (black horizontal midline) and IQR (error bars) are marked. Red horizontal lines compare lncRNAs, or PCGs, between fractions: adjusted *p*-value < 0.05 (*), or < 0.0001 (**)

RNA-seq analysis of nucleoplasmic and chromatin-bound RNA identified more than 3000 subcellular compartment-biased lncRNAs, 99% of which were significantly enriched in the chromatin fraction (3028 vs. 29 lncRNAs, Fig. 4a; Table S2B). Preferential enrichment in the chromatin fraction was also seen for 92% of more than 7000 other lncRNAs that did not meet our stringent criteria (adjusted *p* < 0.001) for differential enrichment between fractions (Figure S4B, Table S2B). In contrast, PGCs were more likely to be significantly enriched in the nucleoplasm than in chromatin (Fig. 4a, Figure S4A), suggesting PCG transcripts are rapidly released from their chromatin-associated transcriptional complexes. Indeed, the magnitude of the compartment bias was significantly lower for chromatin-enriched PCG transcripts than for chromatin-enriched lncRNAs (Figure S4C), consistent with the efficient release of PCG but not lncRNA transcripts to the nucleoplasm following transcription. Transcript maturity was significantly higher for all classes of PCGs, but not for lncRNAs, in the nucleoplasm than in the chromatin fraction, consistent with this model (Figure S4E). Finally, the nucleoplasmic and chromatin enriched PCGs were enriched for distinct biological processes: top enriched terms describing the most highly nucleoplasm-biased PCGs include transmembrane helix, secreted, extracellular matrix, cadherin, blood coagulation and immunity (Table S2D); while the most highly chromatin bound-biased PCGs were most highly enriched for the terms synapse, sequence-specific DNA binding, ion channel activity, and multicellular organism development (Table S2E).

### Impact of polyA selection on lncRNA profiles

Many more lncRNAs were significantly enriched in non-polyA-selected chromatin-bound RNA (which includes both poly-adenylated and non-poly-adenylated transcripts), as compared to the polyA-selected fraction (*n* = 2074 vs. *n* = 844). In contrast, PCGs were more commonly enriched in the polyA-selected fraction (*n* = 3183 vs *n* = 2130) (Fig. 4b, Table S2C, Figure S5A-S5D). Thus, PCGs have a greater tendency than lncRNAs to be poly-adenylated when bound to chromatin. Further, transcript maturity was significantly higher for the chromatin-bound PCG transcripts enriched in the polyA-selected compared to those enriched in the non-polyA-selected fraction (Figure S5E), consistent with the association of poly-adenylation with transcript maturation [43]. In contrast, the tendency for lncRNAs to be enriched in the non-polyA-selected fraction is consistent with splicing being delayed or incomplete for lncRNAs [44]. Chromatin-bound PCG transcripts enriched in the non-polyA-selected fraction had a significantly longer mean gene length and intron length as compared to PCGs transcripts enriched in the polyA-selected fraction (Fig. 4c, d), consistent with these PCGs requiring longer times for completion of transcription and/or processing prior to poly-adenylation. Longer gene lengths were also seen when comparing nuclear-enriched to cytoplasm-enriched PCGs (Figure S3C), but not when comparing chromatin-enriched to nucleoplasm-enriched PCGs (Figure S4F). Finally, top enriched terms for the genes most highly enriched in the polyA-selected chromatin-bound fraction include ribosomal protein, oxidative phosphorylation/mitochondria, non-alcoholic fatty liver disease, and mRNA-splicing (Table S2F); while the top enriched terms for the non-polyA-selected chromatin-bound PCGs included nucleosome assembly (primarily histone genes, whose transcripts are not poly-adenylated [45]), metal binding/zinc finger proteins, Pleckstrin homology domain, and DNA-binding (Table S2G).

We observed a distinct cluster of chromatin-bound transcripts, comprised of 506 lncRNAs and 26 PCGs, with > 64-fold higher relative levels in the non-polyA-selected than in the polyA-selected fraction (Fig. 4b, green box). All of these lncRNAs show their highest expression in the chromatin-bound, non-polyA-selected fraction across all four treatment groups (Fig. 4e), consistent with these being lncRNA transcripts that undergo little or no poly-adenylation. Similarly, 23 of the 26 PCGs were most highly expressed in the non-polyA-selected fraction (Fig. 4f), including several histones RNAs, which as noted, are not poly-adenylated [45]. Other PCGs in this group include the gap junction protein Gja6 and two beta-cadherin protogenes (Pcdhb11, Pcdhb21) and three zinc-finger genes (Rnf148, Zfp691, Zfp804b).

### Increased sensitivity for lncRNA detection in chromatin fraction

Comparison of lncRNA levels across fractions revealed more than a 10-fold increase in the number of lncRNAs expressed (fragments per kilobase length per million mapped sequence reads (FPKM) > 1) when going from the cytoplasm (*n* = 388) to the nucleus (*n* = 983) or nucleoplasm (*n* = 958) to the chromatin-bound fractions (*n* = 3936, *n* = 4610) (Fig. 4g). Median lncRNA levels also increased significantly across the five fractions, with the sensitivity for lncRNA detection increasing 32-fold in chromatin-bound non-polyA RNA (median expression = 1.69 FPKM; IQR, 0.84 to 4.22) as compared to total nuclear RNA (median expression = 0.052 FPKM, IQR, 0 to 0.28) (adjusted *p*-value < 0.001) (Fig. 4g). In contrast, PCGs did not show a major subcellular fraction-dependent increase in expression (Fig. 4h).

### Discovery of sex-biased and TCPOBOP-responsive lncRNAs

**Fig. 5:**
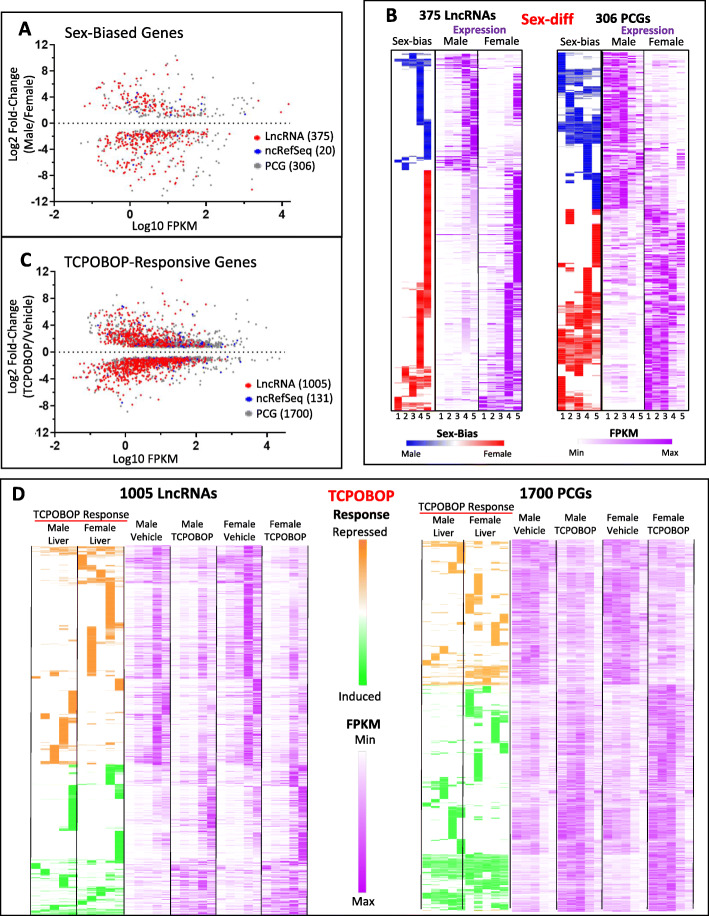
Cell fraction-dependence of sex-biased and TCPOBOP-responsive genes. **a** Genes showing differential expression between male and female liver (edgeR adjusted p-value cutoff of 0.05) in at least one of the five subcellular fractions, based on Table S3A, columns D and E. Gene totals: 123 male-biased and 252 female-biased lncRNAs, 11 male-biased and 9 female-biased non-coding RefSeq genes, and 134 male-biased and 172 female-biased PCGs. **b** Sex-bias and expression in both sexes across all 5 subcellular fractions (left to right, Cytoplasm, Nucleus, Nucleoplasm, Chromatin Bound, Chromatin Bound non-PolyA selected) (Table S3A, columns AA-AT). Expression of each gene (row) is normalized to the highest expression (or strongest sex-bias) of the gene across all fractions. **c** Genes showing differential expression between vehicle and TCPOBOP-treated liver (edgeR adjusted p-value cutoff of 0.05) in at least one of the five subcellular fractions, based on Table S3B, columns D and E. In **a** and **c**, for any gene that is significantly biased in more than one fraction, the fraction with the maximum FPKM and its corresponding fold-change is graphed. Gene totals: 411 up and 594 down regulated lncRNAs, 69 up and 62 down regulated non-coding RefSeq genes, and 1035 up and 665 down regulated PCGs. **d** TCPOBOP responsiveness and expression in both male and female liver is shown across all 5 subcellular fractions (left to right, as in panel **b**) (Table S3B, columns AG-BT). Expression of each gene (row) is normalized to the highest expression or TCPOBOP responsiveness of that gene in a single condition and fraction. In many cases, significant differential expression was seen in only one subcellular fraction for both sex-biased genes (**b**) and TCPOBOP-responsive genes (**d**); in many cases, the same trends were apparent but lacked statistical significance due to very low expression in other fractions and/or variation between biological replicates (Table S3)

Given the striking enrichments of distinct sets of lncRNAs in each subcellular fraction and the increased sensitivity of lncRNA detection seen in chromatin-bound RNA, we used our datasets to discover novel regulated lncRNAs. Differential expression analysis of untreated male versus female liver identified 701 sex-biased genes, 96.6% of which were autosomal, and including 375 sex-biased lncRNAs and 20 other non-coding RefSeq genes (Fig. 5a, Table S3A). 94% (352/375) of the lncRNAs showed sex-biased expression in one or both chromatin-bound fractions, whereas only 18% showed sex-biased expression in the cytosol or nucleoplasm (Table S3A, Fig. 5b). We also identified large numbers of lncRNAs that were induced or repressed by the CAR agonist ligand TCPOBOP [40] in male or female liver (Table S3B and Fig. 5c; 1005 lncRNAs and 131 other noncoding RefSeq genes, including 26 miRNAs). Many of these lncRNAs and PCGs responded to TCPOBOP in one sex only (Fig. 5d, left two columns of each gene set), consistent with our prior findings [22]. 81% of the 1005 lncRNAs regulated by TCPOBOP were responsive in one or both chromatin-bound fractions (Table S3B), highlighting the advantages of RNA-seq analysis of chromatin-bound RNA for discovery of condition-specific, transcriptionally-regulated lncRNA genes.

### smFISH analysis of lncRNA localization

**Fig. 6:**
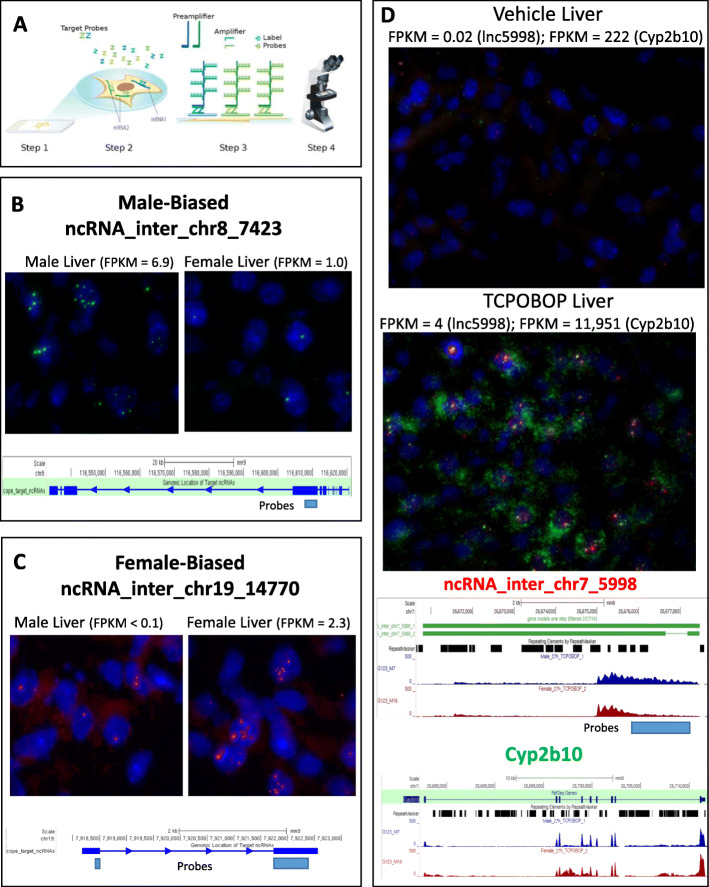
smFiSH analysis of sex-biased and TCPOBOP-responsive lncRNAs. **a** Schematic describing ACD’s RNAScope technology. ZZ probes are hybridized to the gene of interest, then pre-amplifier and amplifiers build in a tree-and-branch manner to amplify the probe/label signal up to 8000-fold, enabling visualization of single RNA molecules. Visualization of expression for: **b** *lnc7423*, a male-biased lncRNA; **c** *lnc14770*, female-biased lncRNA, using ZZ probes designed to a small region of each target lncRNA; and **d** *lnc5998*, a TCPOBOP-inducible lncRNA, and the nearby PCG, *Cyp2b10*, using ZZ probes designed to a small region of *lnc5998*, and across the exonic structure of *Cyp2b10* for vehicle control and 51 h-TCPOBOP-exposed female mouse liver slices. Shown are expression levels (FPKM values, from nuclear RNA-seq, for each gene). See Table S1D for lncRNA genomic coordinates and annotations, Figure S6 for quantitative analysis of smFISH data for all four genes in both sexes, and Table S7 for FPKM values for each gene in each subcellular fraction

We used smFiSH (Fig. 6a) [46] to localize two sex-biased lncRNAs in mouse liver slices. lnc7423 (Fig. 2b), which shows significant male-biased expression, was visualized at several copies per cell in male liver, while in female liver, only a few cells showed expression (Fig. 6b; Figure S6A, S6B), consistent with its strong, male-bias expression seen in the nuclear fractions by RNA-seq (Table S3A). lnc14770, a female-biased lncRNA, was detected at < 1 copy per cell in male liver, but in female liver, five or more copies were seen in some cells, although many cells apparently had only one copy (Fig. 6c; Figure S6C, S6D). Both sex-biased lncRNAs were almost exclusively nuclear and appeared as focal dots, consistent with tight chromatin binding. Based on our RNA-seq data, *lnc7423* is 4–6-fold enriched in the chromatin fraction in both sexes, whereas the female-biased *lnc14770* only showed a significant nuclear bias in female liver (22-fold; Table S7). We also visualized expression of *Cyp2b10* and the divergently transcribed (5.1 kb upstream) *lnc5998*, both of which are highly induced by TCPOBOP [22]. In untreated male liver, *Cyp2b10* expression was very low, with a few RNA molecules detected in the cytoplasm, whereas expression of *lnc5998* was essentially undetectable. Following TCPOBOP exposure, large dense clouds of Cyp2b10 RNA surrounded each nucleus, consistent with the high induction of this RNA seen by RNA-seq and its association with endoplasmic reticulum-bound polysomes. *Cyp2b10* transcripts showed 3-fold nucleoplasmic bias in TCPOBOP treated livers from both male and female mice (Table S7). Very strong induction of *lnc5998* transcripts was also apparent, which in contrast to *Cyp2b10* transcripts, were more concentrated in nuclei, consistent with *lnc5998* showing its highest expression in nuclear and chromatin-bound fractions from both male and female TCPOBOP-treated liver (Fig. 6d; Figure S6E, S6F). Bright, coincident smFISH spots for lnc5998 and Cyp2b10 RNA were observed in many nuclei, indicating co-localization of the transcripts at the site of transcription.

### Integration with prior liver lncRNA expression datasets

**Fig. 7:**
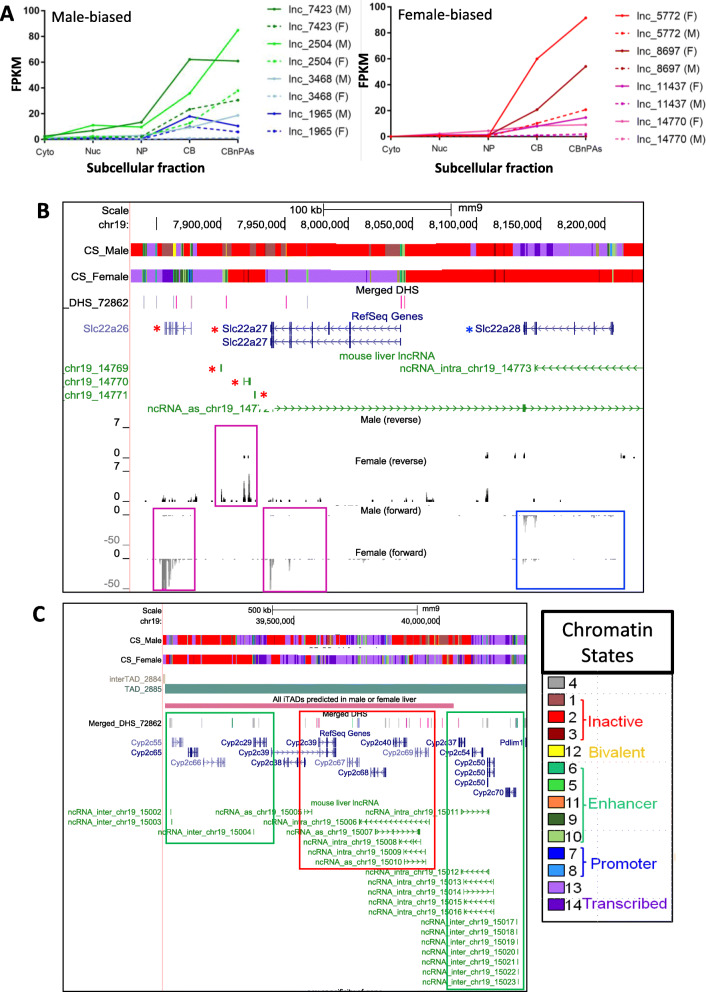
Sex-biased and TCPOBOP-responsive lncRNAs: select examples. **a** Robust male-biased (left) and female-biased lncRNAs (right) (Table S4A, column J) and their expression levels across five subcellular compartments (data based on Tables S2A-S2C). Solid lines indicate expression level in livers of the dominant sex, and dashed lines indicate expression in the opposite sex. Also see Table S3A. **b** and **c** UCSC genome browser screen shots highlighting individual sex-biased genes. The first two tracks in each panel describe the chromatin state (CS) in untreated male and female liver (red = inactive state, green = enhancer state, purple = actively transcribed state; see key of all 14 chromatin states at bottom right, based on [47]). Also shown are tracks indicating the TAD structure (horizontal bar, in **c**), as marked, followed by the Merged DHS track, which marks DHS that are female-biased (pink vertical bars), male-biased (blue bars) and sex-independent (gray bars). Tracks presenting RefSeq gene structures (blue) and lncRNA structures (green) follow. **b** Three female-biased lncRNAs and two female-biased PCGs (red asterisks, pink boxes), all within a TAD that also contains the male-biased gene *Slc27a28* (blue box) (see Table S6A). Shown at the bottom are four tracks with normalized RNA-seq reads in the nucleoplasmic fraction for vehicle-treated male and female liver on the forward and reverse strands, as indicated. **c** TAD containing many *Cyp2c* genes and lncRNAs. The 4th track from top shows a predicted intra-TAD (iTAD) loop (long pink horizontal bar) that is found in female liver only. The genomic region shown is divided into 3 regions whose genes are either up regulated (green boxes) or down regulated (red box) by TCPOBOP exposure (see Table S6B). The first segment includes 5 up-regulated genes, including *Cyp2c55*, which is induced by TCPOBOP > 200-fold. The strongest of the two induced lncRNAs in this segment, *lnc15004*, is also induced 200-fold and is a robust TCPOBOP-responsive lncRNA. The middle segment contains 4 lncRNAs and 5 PCGs, all of which are repressed by TCPOBOP, and the third segment contains three PCGs and two lncRNAs up regulated by TCPOBOP, including the robustly responsive lncRNA, *lnc15014* (80-fold induction)

We integrated the above sets of regulated lncRNAs with prior, published datasets to help identify lncRNAs that are strong candidates for regulatory roles in the liver. We designated 49 lncRNAs as robust sex-biased genes, based on their significant sex-biased expression in at least 2 of 5 subcellular fractions analyzed here (Table S3A) and in at least 5 of 11 prior liver RNA-seq datasets (Table S4A). These 49 lncRNAs are highly expressed and strongly sex biased: 40 show a maximum FPKM > 2, and 41 show a > 4-fold sex-bias in at least one subcellular fraction. Figure 7a presents expression data in both sexes across subcellular fractions for eight of these lncRNAs, and highlights the large increases in relative lncRNA levels, and hence the increased sensitivity for detection, in the chromatin-bound fractions. A large majority (86%) of the robust sex-biased lncRNAs showed a significant change in expression in livers of hypophysectomized mice, where the growth hormone signaling required for sex-biased gene expression in liver is ablated  [32]. Furthermore, 19 of the 49 lncRNAs exhibited developmental changes in expression in male mouse liver during the transition from the pre-pubertal stage to young adulthood [26], which has been linked to the sex-dependent expression of key transcription factors and sex-biased genes involved in specialized liver functions [48, 49]; these lncRNAs may contribute to the post-pubertal changes in expression commonly seen for sex-biased PCGs in male liver. Finally, 33 of the 375 sex-biased lncRNAs identified here showed significant sex-biased expression in few or none of the prior datasets (Table S4B). Many of these lncRNAs (29/33) were maximally expressed in one of the chromatin-bound fractions, which may explain why they were not detected previously by RNA-seq analysis of total liver or liver nuclear RNA.

We also identified 96 robust TCPOBOP-responsive lncRNAs (Table S5A, S5B), many being highly expressed (79 have maximum FPKM > 2) and highly responsive to TCPOBOP (74 show > 4-fold maximum response). Many also responded to other chemicals that dysregulate gene expression in the liver, including phenobarbital (*n* = 45 lncRNAs), acetaminophen (*n* = 27) and agonists of PPARA (either WY14634 or fenofibrate) (*n* = 33). In contrast, 334 of the 1005 TCPOBOP-responsive lncRNAs identified here were identified as responding in at most one of the five prior TCPOBOP-treated datasets. Seventy of these 334 novel TCPOBOP-dysregulated lncRNAs also responded to at least one of four other chemicals examined (phenobarbital, acetaminophen, WY14634 and fenofibrate), and 282 (84%) were maximally expressed in one of the chromatin-bound fractions, which may explain why they were not identified previously. The novel TCPOBOP-inducible lncRNAs include *lnc4278*/*Dancr*, *lnc14777/Snhg1*, and *lnc10895/Snhg10*, which promote hepatocellular carcinoma through their actions as miRNA sponges [50–52], and *lnc733/Gas5*, which is also a miRNA sponge and acts to inhibit liver fibrosis [53].

### LncRNAs as potential regulators in *cis*

Many lncRNAs function as regulators in *cis*, whose transcription regulates nearby PCGs by a variety of mechanisms [8, 54, 55]. To identify sex-biased and TCPOBOP-responsive lncRNAs that may serve as *cis*-regulators, we considered lncRNAs co-localized with PCGs within TADs [56]. TADs are megabase-scale chromatin loops organized by interactions between CTCF and the cohesin complex; they loop together relatively distant regions of chromatin, allowing regulatory elements and their bound factors, including chromatin-tethered lncRNAs on one end of a TAD to regulate in *cis* genes located on the other end of the TAD [57]. Using TAD definitions for mouse liver [57] (Table S6C), we identified 36 TADs that contain at least one strongly sex-biased lncRNA (> 4-fold sex difference in expression) and harbor at least one sex-biased non-lncRNA gene (Table S6A). These 36 TADs encompass 93 sex-biased lncRNAs, 4 sex-biased non-coding RefSeq genes, and 71 sex-biased PCGs, which serve as candidates for *cis* regulation. Thirty of the 93 lncRNAs are within 13 TADs that each contain at least one gene of the opposite sex bias, and hence are candidates for negative regulation.

One example, is a genomic region defined by a gap between two TADs (inter-TAD region, 960 kb long) that contains three female-biased lncRNAs, and two female-biased and one male-biased PCG from the *Slc22a* family (Fig. 7b, red and blue asterisks). All three lncRNAs show strongly female-biased expression (up to 66-fold) with FPKM values as high as 4.5–7.5 in the nucleoplasm or in chromatin (Tables S2A-S2C). The three lncRNAs are located between the two female-biased *Slc22a* genes in a region with multiple female-biased DNase hypersensitive sites (DHS), suggesting the entire genomic region is regulated as a single unit (Fig. 7b). In contrast, the region encompassing the male-biased *Slc22a28*, located > 100 kb upstream of the female-biased genes, is devoid of DHS sites; however, that genomic region is characterized by an active chromatin state in male but not female liver (Fig. 7b, top two tracks). *Slc22a28* could be regulated by the one male-specific DHS found far upstream of *Slc22a28* (342 kb away) but in the same inter-TAD region; alternatively, the female-biased lncRNA(s) could act via a looping mechanism to silence *Slc22a28* expression in female liver, resulting in the observed male-biased expression.

We also identified 211 TADs that contain at least one TCPOBOP-responsive lncRNA (fold change > 4) and at least one TCPOBOP-responsive non-lncRNA; together, they comprise a total of 484 lncRNAs, 418 PCGs and 28 non-coding RefSeq genes (Table S6B). One example is a TAD that encompasses 6 TCPOBOP-regulated lncRNAs (4 induced, 2 repressed) and 13 TCPOBOP-regulated *Cyp2c* gene subfamily PCGs (8 induced, 5 repressed) (Fig. 7c). This TAD encompasses 3 segments; the first and the third segments contain TCPOBOP-induced genes, and the middle segment contains TCPOBOP-repressed genes. This arrangement suggests the TAD is divided into 3 insulated regions in TCPOBOP-exposed liver, perhaps separated by intra-TAD loops ('sub-TADs') [57]. There is evidence for an intra-TAD loop in untreated female but not male liver [58] that encompasses the first 2 segments but excludes the third (Fig. 7c, 4th track, red horizontal bar). Of note, a majority of the genes in the TCPOBOP-repressed middle segment are more highly responsive to TCPOBOP in female liver, where 4 of 5 genes show female-biased expression in vehicle-treated liver. This female-specific intra-TAD loop [58] could allow the strong induction of lnc15004 to lead to repression of these genes in TCPOBOP-treated female liver.

### Divergently transcribed lncRNAs showing sex-biased expression

**Fig. 8:**
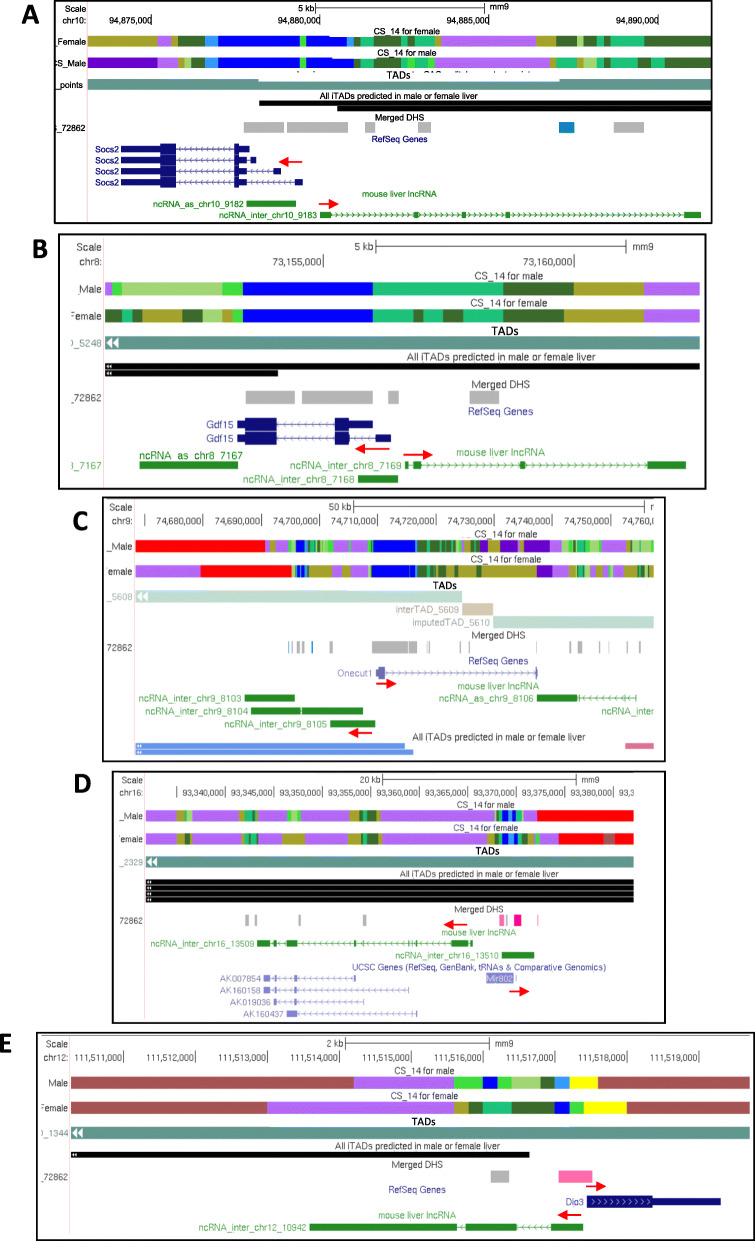
Divergently transcribed lncRNA-PCG genes of interest. Shown are UCSC genome browser screen shots, as described in Fig. 7, highlighting five divergent lncRNA-PCG gene pairs, whose direction of transcription is marked by red arrows. See data in Table S4A, column AJ, and Table S5A, column AX. **a** Divergent sex-biased and TCPOBOP-responsive gene pair, *lnc9183* and *Socs2*. **b** Divergent TCPOBOP-responsive gene pair, *lnc7169* and *Gdf15*. These genes fall within a sex-independent intra-TAD loop (black) with several sex-independent DHS (gray). **c** Divergent TCPOBOP-responsive gene pair, *lnc8105* and *Onecut1* (*Hnf6*), both of whose TSS are in the same male-biased intra-TAD loop (last track, light blue). **d** Divergent TCPOBOP-responsive gene pair, *lnc13509* and *Mir802*. These genes fall within a sex-independent intra-TAD loop (black) with several female-biased DHS (pink) in the promoter regions of both genes. **e** Divergent TCPOBOP-responsive gene pair, *lnc10942* and *Dio3*. These genes are excluded from the predicted intra-TAD loop nearby (black) and their promoters are both within a shared female-biased DHS region (pink)

Divergent lncRNAs are defined as lncRNAs with a TSS < 5 kb from the TSS of a non-overlapping PCG transcribed from the opposite strand. They are frequently adjacent to regulatory genes, whose expression or activity is controlled in cis by the divergently transcribed lncRNA [54, 59]. Accordingly, one can infer the biological function of a divergent lncRNA from that of its neighboring gene. We identified six sex-biased lncRNAs that are divergently transcribed from a sex-biased PCG (Table S4A, column AJ). One of these divergent gene pairs encodes SOCS2, a STAT5-induced inhibitor of STAT5 signaling [60, 61] that shows 3 to 5-fold female-biased expression (Table S6A). SOCS2 and other SOCS family proteins are negative feedback regulators of STAT5-dependent growth hormone signaling [61] and are proposed to contribute to the inhibitory effects of persistent growth hormone stimulation on STAT5 signaling in female liver [62]. SOCS2 also inhibits metastasis in hepatocellular carcinoma [63], a male-predominant disease [64, 65]. *Lnc9183* is divergently transcribed from *Socs2* (Fig. 8a) and showed 9-fold female-biased expression (FPKM = 13 in chromatin-bound RNA; Table S4A). *Socs2* has several isoforms, and the major transcript in the cytoplasmic, nuclear and nucleoplasmic fractions has its TSS within an intra-TAD structure together with the TSS of its divergent, sex-biased lncRNA partner. This genomic organization may insulate lncRNA-driven regulation of *Socs2* in female liver from other genes elsewhere in the TAD that are not sex-biased. Interestingly, *Socs2* and the divergently transcribed *lnc9183* are both TCPOBOP-responsive (Table S6B), as are several other, more distant lncRNAs in the same TAD (*lnc9185* and *lnc9178*), which could impact STAT5 regulation of its many downstream sex-biased gene targets in male and female liver [66].

### Divergent transcription of TCPOBOP-responsive lncRNA and PCG gene pairs

We also identified 51 divergently transcribed, TCPOBOP-responsive lncRNA-PCG pairs in either male or female liver (Table S5A, column AW). Four examples are described below.

1) *Lnc7169* is divergently transcribed from *Gdf15* (Fig. 8b), a stress response cytokine [67]. *Lnc7169* has both rat and human orthologs, and both it and *Gdf15* are strongly induced by TCPOBOP (up to 42-fold and 18-fold, respectively; Table S6B). The rat ortholog maps to a gene network involving glycerolipid metabolism, and its expression in liver is dysregulated by 16 of 27 xenochemicals tested [27]. Gdf15 impairs progression of non-alcoholic fatty liver disease in obese mice by enhancing fatty acids oxidation [68, 69] and its deficiency promotes high fat diet-induced obesity [70] and exacerbates liver injury induced by chronic alcohol and carbon tetrachloride exposure [71]. We hypothesize that the multi-xenobiotic-responsive *lnc7169* has a role in regulating these beneficial *Gdf15*-dependent hepatic disease-related processes.

2) *Lnc8105* is divergently transcribed from *Onecut1/Hnf6*, a major liver-enriched transcription factor implicated in the expression of many liver-specific genes, including sex-biased genes [23, 66, 72] (Fig. 8c). TCPOBOP strongly down regulated both genes, in both male and female mouse liver. This repression of Onecut1/Hnf6 RNA was observed in the chromatin fraction, indicating a decrease in of *Onecut1/Hnf6* transcription, but surprisingly, it did not result in a decrease in cytoplasmic Onecut1/Hnf6 RNA (Table S3B). The impact of this repression by TCPOBOP of *Onecut1/Hnf6* and its divergently transcribed, chromatin-associated lncRNA on the many downstream transcriptional targets of HNF6 in mouse liver [72] is unknown.

3) *lnc13509* (*1810053b23Rik*) is transcribed divergently from *miR802* (Fig. 8d). Both genes are strongly induced by TCPOBOP (> 15-fold; Table S6B). *Lnc13509* is highly expressed in fetal liver and then repressed during development [26]. It has a close human ortholog (57% identity) [27] and its rat ortholog is induced by multiple xenobiotics and is in a co-expression network of xenobiotic-responsive genes enriched in transcription factors [27]. We hypothesize that the induction of *lnc13509* impacts various biological and pathological responses regulated by *miR802*. Elevated expression of *miR802* is seen in type-II diabetes [73] and in livers of high fat diet-fed mice, where it impairs glucose metabolism by silencing the transcription factor HNF1B and by increasing oxidative stress [74–76]. Further, *miR802* shows female-biased expression in mouse liver; it preferentially represses many male-biased mRNAs and increases levels of female-biased mRNAs in female liver [77] and has been associated with regulation of glucose and lipid metabolism [76].

4) *Lnc10942/Dio3os* is repressed up to 20-fold in mouse liver by agonists of the nuclear receptor CAR but was induced 5-fold by PPARA agonists (Table S5A, Table S5C). Its rat ortholog is responsive to 13 out of 27 xenochemical exposures in rat liver [27]. Repression of *Dio3os* has been linked to increased cell proliferation, and its repression is a biomarker for inflammatory bowel disease [78, 79]. *Lnc10942/Dio3os* is divergently transcribed from *deiodinase-3* (*Dio3*) (Fig. 8e), a seleno enzyme that inactivates the thyroid hormones T3 and T4 [80]. Systemic thyroid hormone inactivation occurs upon activation of CAR in mouse liver, and is also a physiological response that limits weight loss upon fasting/caloric restriction [81, 82]. Further, *Dio3* undergoes translational repression in models of drug-induced inflammation and hepatotoxicity. Given the opposite effects of CAR vs. PPARA activation on the expression of *lnc10942/Dio3os*, this lncRNA may contribute to some of the differing physiological effects of CAR vs PPARA activation in liver. *Lnc10942/Dio3os* may regulate responses to hepatic stressors, such as fasting, high fat diet, and the need to regulate thyroid hormone levels by *Dio3*.

## Discussion

Global patterns of gene expression, maturation and subcellular localization were determined for thousands of liver-expressed lncRNAs and PCGs using a fractionation protocol that allowed us to isolate, from the same individual mouse liver, cytoplasmic and nuclear RNA, as well as a soluble nucleoplasmic RNA fraction and RNA tightly bound to chromatin. Transcripts enriched in the chromatin-bound fraction were the least mature, as indicated by a high fraction of sequence reads mapping to introns, while cytoplasmic transcripts were the most mature. In contrast to PCGs, lncRNAs were highly enriched in the nucleus, and specifically in the chromatin-bound fraction, rather than the nucleoplasm. Furthermore, many lncRNAs were most highly expressed in non-polyA-selected chromatin-bound RNA, consistent with findings in human cell lines that lncRNAs, as well as chromatin-enriched RNAs, are less poly-adenylated than mRNAs [31]. The increased sensitivity for lncRNA detection in chromatin-bound RNA enabled us to identify 375 lncRNAs showing sex-biased expression, as well as 1005 lncRNAs that were significantly induced or repressed in livers from mice treated with the CAR agonist TCPOBOP, many of which were not identified in earlier work where nuclear but not chromatin-bound RNA was analyzed [22, 26]. Finally, we identified lncRNAs associated with divergently transcribed lncRNA-PCG pairs, many of which are anticipated to have regulatory functions [59], as well as candidates for *cis*-acting lncRNAs [55, 83], based on their presence in the same TAD [56, 57] as correspondingly responsive, or in some cases oppositely responsive PCGs.

We used normalized intronic to exonic read densities (IO/EC ratio) to assess the extent of transcript splicing in each cell fraction. This approach is similar to calculating the degree of splicing based on exonic base coverage divided by base coverage over the entire transcript [30], and for the many lowly expressed lncRNAs, it is much more sensitive than an alternative method that directly calculates splicing based on completed splice junction reads [44]. Using our approach, we found splicing was comparatively low in the chromatin-bound nuclear fraction for both lncRNAs and PCGs, independent of polyadenylation selection. This suggests that lncRNA and PCG splicing initially proceed in a similar manner, with polyA addition preceding, or occurring at the same time, as splicing [44, 84]. Further, while PCG transcripts apparently became increasingly more mature in moving from chromatin to the nucleoplasm and then on to the cytoplasm, lncRNA transcripts showed less extensive splicing than PCG transcripts in the nucleoplasmic, nuclear and cytoplasmic compartments, despite the presence of multiple splice sites junctions in many lncRNA transcripts. This finding is consistent with studies indicating that lncRNAs do not necessarily require splicing to be functional [44, 85]. However, we cannot rule out the possibility that some of the differences between PCG transcript and lncRNA maturation indicated by our data are due to a more rapid loss of PCG transcripts with retained introns via nonsense-mediated decay [86], rather than more efficient mRNA processing per se. Finally, we note that splicing completion might not be required for many lncRNAs, if they in fact serve an evolutionary role, rather than a specific functional role, as was recently proposed [87].

Comparing relative transcript levels (relative transcript concentrations) between subcellular fractions, we found that many liver-expressed lncRNAs are highly enriched in the nuclear and chromatin-bound fractions and are substantially depleted from the cytoplasm and the nucleoplasm. Overall, 92% of liver lncRNAs showing significant differential enrichment between cytoplasm and nucleus were enriched in the nucleus, and 99% of lncRNAs differentially expressed between the chromatin fraction and nucleoplasm were chromatin-enriched. This strong apparent enrichment of lncRNAs for chromatin binding likely involve multiple mechanisms, ranging from enhanced lncRNA degradation in the nucleoplasm or cytoplasm to active lncRNA retention in the chromatin fraction. One mechanism could relate to the inefficiency of splicing-coupled lncRNA export due to weak internal splicing signals and the associated increase in Pol II occupancy on lncRNA as compared to PCG introns [88]; however, we did not find a significant association between maturation of lncRNA transcripts and their enrichment for chromatin binding. Other features of lncRNAs that likely contribute to the tight chromatin binding seen in this study include the presence of specific *cis*-elements that mediate nuclear retention, such as repeat insertion domains, SINE-derived localization elements [89, 90] and motifs associated with U1 snRNP binding [91]. Chromatin binding is likely to be an important driver of many nuclear lncRNA functions, including direct or indirect regulation of chromatin states and gene transcription. Chromatin-bound lncRNAs may act in *cis* at sites in the genome close to their transcription [55], but some may transit through the nucleoplasm and be *trans*-acting [8]. Indeed, many of the lncRNAs enriched in the chromatin fraction were also present in the nucleoplasm at significant concentrations, which could allow them access to multiple *trans* sites within the nucleus. Finally, we note that the subcellular fraction enrichments presented here, which are based on relative transcript concentrations, do not equate with absolute localizations, as they do not take into account differences in the total amount of polyA RNA in each fraction [21]. However, relative and absolute RNA localization values are likely to be qualitatively the same for many of the nuclear-enriched and chromatin-enriched liver lncRNAs characterized here, whose transcripts often showed very large (>10-fold) differential concentrations between fractions (Fig. 3a).

A majority of the liver lncRNAs we characterized appear to be poly-adenylated, insofar as they were recovered from polyA-selected RNA. Nevertheless, by comparing a polyA-selected to a non-polyA-selected chromatin fraction, we identified many chromatin-bound lncRNAs that were enriched in non-polyA-selected RNA. Moreover, a distinct subset comprised of 506 chromatin-bound lncRNAs, as well as 26 PCGs, was apparently not poly-adenylated, insofar as they showed > 60-fold greater abundance in the non-polyA-selected fraction (Fig. 4e). Many chromatin-enriched lncRNAs are under-spliced compared to mRNAs [31] and yet appear to be functional despite incomplete splicing and/or poly-adenylation [44, 90]. Several subclasses of lncRNAs are not spliced, including very long intergenic lncRNAs, macro lncRNAs, and circular lncRNAs [85, 92]. Computational methods have been developed to predict lncRNA subcellular localization based on features such as splicing efficiency and the presence of certain k-mer sequences, specific binding motifs, and genomic characteristics [88, 93]. Current methods are ~ 75% accurate in predicting nuclear versus cytoplasmic localization of well characterized lncRNAs [93], and efforts at further refinement will benefit from experimentally validated datasets such as those presented here.

PCG transcripts were more likely than lncRNAs to show both a cytoplasmic (vs. nuclear) bias and a nucleoplasmic (vs. chromatin-bound fraction) bias. Moreover, PCGs enriched in the cytoplasm were more extensively spliced in both the cytoplasm and the nucleus than their nuclear-enriched PCG counterparts. The same pattern was seen when comparing nucleoplasmic and chromatin-bound PCG transcripts, consistent with their localization bias largely being driven by transcript maturation. Indeed, chromatin-bound PCG transcripts showing a bias for the non-polyadenylated fraction were encoded by longer genes with a higher intronic content than transcripts biased toward the polyA-selected fraction; their enrichment in the non-polyadenylated fraction can thus be explained by the longer times required for gene transcription and splicing as compared to shorter, lower intronic content PCGs. mRNA export to the cytoplasm is facilitated by the completion of mRNA processing, and nuclear-retained mRNAs often contain introns [43, 94–96]. Nuclear retention of mRNAs can be permanent, but may also be reversible in response to cell stressors [94]. These events are thought to aid in the stress response by stockpiling mRNAs for rapid release [97] and may also minimize fluctuations in protein levels due to bursty transcription [98].

Finally, we identified 375 lncRNAs showing sex-biased expression, as well as 1005 lncRNAs responsive to the CAR agonist ligand TCPOBOP. Many of these lncRNA gene regulatory responses were observed in the chromatin-bound RNA fractions, consistent with both processes being regulated at the transcriptional level. For many lncRNAs, the highest level of expression was often seen in the chromatin fraction, which increased the sensitivity for their detection and helps explain why a subset of these regulated lncRNAs were not identified in earlier whole liver or total nuclear RNA-seq analyses. Many of the sex-biased and TCPOBOP-responsive lncRNAs may be *cis*-acting, based on their location within the same TADs as similarly regulated, or in some cases oppositely regulated PCGs, which is expected to facilitate TAD-based promoter-enhancer interactions, including lncRNA-PCG interactions [58]. Overall, 25% of the sex-biased lncRNAs were located in TADs with other sex-biased genes. Similarly, 48% of TCPOBOP-responsive lncRNAs were in TADs with other TCPOBOP-responsive genes, giving them the potential to act in *cis*. We also identified 6 cases where sex-biased lncRNAs are divergently transcribed from correspondingly sex-biased PCGs, and 51 cases of divergently transcribed TCPOBOP-responsive lncRNA-RefSeq pairs. The presence of close orthologs in rat or human for several of the divergently transcribed, xenobiotic-responsive lncRNAs [27] supports their proposed functional roles in liver responses to foreign chemical exposure. In one example, the strong induction of *lnc7169* by TCPOBOP may contribute to the hepatoprotective effects of CAR activation on high fat diet-induced non-alcoholic fatty liver disease [99, 100] by increasing expression of the divergently transcribed *Gdf15*, a stress response cytokine that is induced by inflammation, acute injury and oxidative stress [67]. In contrast, the very strong induction by TCPOBOP of *lnc13509* may stimulate the divergent transcription of *miR802*, whose expression is elevated in type-II diabetes [73] and in livers of high fat diet-fed mice, where it impairs glucose metabolism and increases oxidative stress [74–76]. Alternatively, *lnc13509* could serve as a hepatoprotective miRNA sponge [101] that depletes *miR802*. These and other putative regulatory lncRNAs may be investigated using a variety of experimental and computational approaches [102, 103], including innovative knockout technologies [36] that may uncover their biological functions and gene targets in the liver.

## Conclusions

We characterized global patterns of expression, maturation and subcellular localization for the mouse liver transcriptome, including more than 15,000 lncRNAs, many of which showed tight binding to chromatin. Sequencing chromatin-bound RNA greatly increased the sensitivity for detecting lowly expressed lncRNAs and enabled us to discover and localize hundreds of novel regulated liver lncRNAs, including lncRNAs showing sex-biased expression or responsiveness to a xenobiotic agonist ligand of the nuclear receptor CAR. Integration of our findings with prior studies identified strong candidates for lncRNAs that regulate a variety of hepatic functions, based on their co-localization within TADs, or their transcription divergent or antisense to PCGs associated with pathways linked to hepatic physiology and disease.

## Methods

### Animal studies

All mouse work was carried out in compliance with procedures approved by the Boston University Institutional Animal Care and Use Committee, and in compliance with ARRIVE 2.0 Essential 10 guidelines [104], including study design, sample size, randomization, experimental animals and procedures, and statistical methods. Male and female CD-1 mice (strain Crl:CD1(ICR)), between 7 and 8 weeks of age, were purchased from Charles River Laboratories (Wilmington, Massachusetts) and randomized into treatment and control groups (*n* = 5 mice in each of four groups). No specific criteria for inclusion or exclusion of animals or data points were set, and experimental follow up used *n* = 3–4 livers, randomly selected from the available n = 5 livers per group, as specified below for each analysis. TCPOBOP (1,4-bis (2-(3,5-dichloropyridyloxy))benzene) (Cat. #sc-203,291, Santa Cruz Biotechnology) was dissolved in DMSO at 7.5 mg/ml and then diluted 10-fold into corn oil, followed by intraperitoneal injection of 4 μl per gram body weight (final dose: 3 mg TCPOBOP and 4 μl of 10% DMSO in corn oil, per kg mouse body weight) at 8 AM (Boston University Lab Animal Care Facility; lights on at 7:30 AM, lights off at 7:30 PM). Mice were euthanized 27 h later (except as noted), at 11 AM, by cervical dislocation under CO_2_, with treatment and control groups processed in parallel for each sex. Liver samples used in this study were flash frozen in liquid nitrogen then stored at − 80 °C, and were prepared by Dr. Hong Ma of this laboratory.

### Isolation of cytoplasmic and nuclear RNA

All pipette tips used were RNase-free and DNase-free (Cat. #76322 series, VWR). RNA was isolated from livers from *n* = 4 mice from each of four treatment groups: vehicle-injected males, 27 h TCPOBOP-treated males, vehicle-injected females, and 27 h TCPOBOP-treated females (Table S1A). The following buffers were prepared fresh daily and kept on ice for up to 2 h: **Base Solution,** 10 mM Tris-Cl, pH 7.4, 146 mM NaCl, 1 mM CaCl_2_ 21 mM MgCl_2_; **Lysis Buffer,** Base Solution containing 0.1% Triton X-100 (Cat. #T8787, Sigma), with 80 U/mL Protector RNase Inhibitor (Cat # 3335402001, Roche) added just prior to use; **ST Nuclei Wash Buffer (ST Buffer),** Base Solution containing 0.01% BSA (Cat. #SRE0036, Sigma), with 80 U/mL Protector RNAse Inhibitor added just prior to use; **BSA Wash Buffer,** 1X PBS containing 2% BSA and 0.02% Tween-20, with 80 U/mL Protector RNase Inhibitor added just prior to use. To minimize premature tissue thawing and RNA degradation, a ~ 250 mg piece of each of four livers per group was placed on dry ice, cut into 2–3 smaller pieces, and stored in an Eppendorf tube on dry ice until ready for further processing. The combined frozen and pre-cut liver pieces were transferred to a 3 mL glass-on-glass Dounce homogenizer on ice containing 1 mL of Lysis Buffer. Keeping the homogenizer on ice, each liver sample was dounced for 10 strokes with pestle A (loose fit) followed by ~ 10 strokes with pestle B (tight fit) until the sample was fully homogenized. Homogenization was performed in under 1 min, while avoiding foaming and splattering of the sample. ST Buffer (1 ml) was then added, pipetted up and down a few times to mix, and the sample was then passed through a 40 μm cell strainer (Cat. # 10199–655, VWR) into a 50 mL conical tube on ice. A second 1 ml of cold ST Buffer was used to rinse the homogenizer and pestles, passed through the same 40 μm cell strainer and then combined with the homogenized sample. The homogenizer and pestles were then washed with Milli-Q water three times before processing the next sample. Homogenized samples were kept on ice until all liver groups were ready to proceed to the next step. Each homogenized sample was divided into two 1.5 ml Eppendorf tubes, which were centrifuged at 500 x g in a swinging bucket centrifuge (Dynac Centrifuge, Clay Adams) for 5 min at 4 °C to pellet the lysed cells. A swinging bucket rotor was used to minimize the shear forces generated using a fixed-angle rotor, which may damage the nuclei. The supernatant was removed from the pelleted nuclei, and a 250 μl aliquot was placed in an Eppendorf tube on ice to extract cytoplasmic RNA. Trizol LS reagent (750 μl) was added immediately to the cytoplasmic fraction, followed by vortexing for a few seconds, then storage at − 20 °C. The pelleted nuclei were gently resuspended in 1 ml BSA Wash Buffer while combining the material from both tubes into one sample, followed by centrifugation in a swinging bucket rotor at 500 x g for 5 min at 4 °C. The supernatant was discarded and the pellet was resuspended in 1 mL BSA Wash Buffer then passed through a 20 μm cell strainer (Cat # 43–50,020-03, PluriSelect), sitting on top of a 50 ml conical tube placed in ice. The strainer and tube were briefly spin at 1500 x g for 15 s. The strained sample was then transferred to two LoBind 1.5 mL Eppendorf tubes (Cat # 022431021, Eppendorf) on ice: one tube with 333 μl was used to isolate nuclear RNA; 667 μl of Trizol LS reagent was immediately added to that tube, which was vortexed for a few seconds then stored at − 20 °C. A second tube with 667 μl of the strained sample was used to fractionate the nuclear RNA, as described below.

### Fractionation of nuclear RNA

This protocol was adapted from [105]. **Fractionation Buffer** (1% Triton X-100, 20 mM HEPES (pH 7.5), 300 mM NaCl, 2 M Urea (Cat #5505UA, Life Technologies), 0.2 mM EDTA, 1 mM DTT, with 250 U/mL Protector RNase Inhibitor) was prepared fresh each day. The nuclei from the nuclei isolation procedure, described above, were re-pelleted at 500 x g in a swinging bucket rotor for 5 min at 4 °C, and the supernatant was discarded. The nuclear pellet was resuspended by gently pipetting in 200 μl Fractionation Buffer, followed by incubation on ice for 10 min then centrifugation at 3000 x g for 2 min at 4 °C. A 180 μl aliquot of the supernatant, corresponding to the nucleoplasmic (NP) fraction, was removed and placed in a clean Eppendorf tube on ice. Immediately, the total volume was brought to 250 μl with Milli-Q water; 750 μl Trizol LS was then added followed by vortexing for a few seconds and storage at − 20 °C. The remaining supernatant (~ 20 μl) was carefully removed without disturbing the pellet and discarded. The pellet (i.e., the insoluble chromatin-bound (CB) fraction) was gently washed twice with 100 μl Fractionation Buffer, taking care to not disturb the pellet, and then centrifuged at 3000 x g for 2 min at 4 °C. The chromatin pellet was then solubilized by digestion for 30 min at 37 °C in 50 μl DNase I solution (1X DNase I buffer containing 0.2 U/μl DNase I [Cat #M6101, Promega] and 0.25 U/μl Protector RNase Inhibitor), with gentle mixing by pipetting every 10 min. The final volume was brought to 250 μl with Milli-Q water. 750 μl Trizol LS was then added, the sample was vortexed briefly and stored at − 20 °C.

### RNA isolation from subcellular fractions using Trizol LS

Frozen samples containing cytoplasmic, nuclear, nucleoplasmic and chromatin-bound RNA suspended in Trizol LS were thawed on ice, and vortexed for 10 s to fully resuspend each sample. Chloroform (isoamyl alcohol free, 0.2 ml) was then added to each sample, followed by vigorous vortexing for 15 s. Each sample was allowed to sit for 2–3 min at room temperature and then spun at 12,000 x g for 15 min at 4 °C. The clear upper, aqueous phase was carefully transferred to a new centrifuge tube, while being careful to not disturb the genomic DNA at the interface. Isopropanol (0.5 ml) was added to each sample followed by vortexing for 10 s. Glycogen (Cat. #AM9510, ThermoFisher) was then added to each sample (1 μg per 20 μl reaction). Samples were vortexed and then incubated for 10 min at room temperature. Samples were centrifuged at 12,000 x g for 20 min at 4 °C and the supernatant was discarded. The RNA pellet was washed with 1 mL of 75% ethanol by vortexing, followed by centrifugation at 7500 x g for 5 min at 4 °C. The ethanol wash was removed and samples were air dried for 5–10 min. Final RNA pellets were resuspended in 20 μl Milli-Q water, quantified on a Qubit instrument using the Qubit RNA HS Assay (Cat. #Q32852, Invitrogen) and stored at − 20 °C.

### qPCR analysis

RNA (0.5 μg) purified from each of four different subcellular fractions, and without polyA selection, was treated with DNase I (Cat. #M6101, Promega) to remove DNA contamination. cDNA was then synthesized using High-Capacity cDNA Reverse Transcription Kit (Cat #4368814, Applied Biosystems). qPCR was performed using primers specific to the RNAs for mouse *18S*, *Cyp2b10*, *Xist*, *Neat1*, *Elovl3* and *pre-Elovl3*, designed using Primer Express and Primer3 software (http://bioinfo.ut.ee/primer3-0.4.0/) (see Table S1A for primer sequences). Quantitative real-time PCR was carried out on a CFX384 Touch Real-Time PCR Detection System (Bio-Rad) using Power SYBR Green PCR Master Mix (ThermoFisher). Normalized linear Ct numbers were computed to determine the relative expression level of each gene across treatments and subcellular fractions to validate the effectiveness of RNA fractionation prior to sequencing library preparation.

### Single molecule imaging of RNAs in liver slices

Single molecule fluorescent in situ hybridization (smFiSH) to frozen mouse liver tissue slices was used to localize individual RNAs. We used RNAScope technology (Advanced Cell Diagnostics, Inc., Newark, CA), which employs a series of up to twenty “ZZ” pairs of 20-mer oligonucleotides hybridized to each RNA transcript as a base for tree-and-branch building, leading to an overall 8000-fold amplification of signal and enabling highly sensitive imaging and localization of single RNA molecules [46]. ZZ probes were designed in cooperation with Advanced Cell Diagnostics staff for three lncRNAs (mouse mm9 genomic coordinates indicated): (1) 20 probes for *lnc7423* across 2 exons, at Chr8(−):116,609,566-116,610,245 (680 bp), and at Chr8(−):116,609,152-116,609,565 (414 bp); (2) 9 probes for *lnc14770* across 2 exons, at Chr19(+):7,918,477-7,918,504 (27 bp) and at Chr19(+):7,921,742-7,922,550 (808 bp); and (3) 20 probes for *lnc5998* over 1 exon, where the majority of the expression is observed, at Chr8(−):26,676,223-26,677,271 (1048 bp). We were unable to design a set of ZZ probes unique for *Cyp2b10* due to its high homology with four other mouse *Cyp2b* subfamily members (*Cyp2b9*, *Cyp2b13*, *Cyp2b19* and *Cyp2b23*). However, we did identify four ZZ probes spread across the exonic structure of *Cyp2b10* that showed low homology with *Cyp2b9* and Cyp2b13 (both liver expressed) but were highly homologous to *Cyp2b19* and *Cyp2b23*, which are not expressed in mouse liver, and in practice gave *Cyp2b10*-specific signals. The high specificity of these four ZZ probes for *Cyp2b10* visualization was verified by the very low smFiSH signal in untreated male liver, where *Cyp2b10* expression is very low. All other ZZ probes were unique to both coding and non-coding regions of the mouse genome.

Fresh livers from male and female CD-1 mice (as described above), controls or treated with TCPOBOP (51 h, 3 mg/kg, i.p.), were frozen in isopentane on dry ice, followed by flash freezing in liquid N_2_ and storage at − 80 °C. Frozen tissue was embedded in OCT medium and stored long-term at − 80 °C. Before cutting tissue slices, frozen tissue was allowed to incubate for 1 h at − 20 °C in the cooling chamber of a Leica CM1950 cryostat. Tissue sections were sliced 15 μm thin and placed on SuperFrost Plus slides (Cat #12–550-15, Fisherbrand) and stored at − 20 °C. Slides were processed using RNAscope Fluorescent Multiplex Reagent Kit v1 for Fresh Frozen Tissue, as described in Advanced Cell Diagnostics documents #320513 and #320293, which list all specialized reagents and equipment used for this protocol, except for 32% paraformaldehyde (Cat #15714-SP, Electron Microscope Science) and Prolong Gold Antifade with DAPI (Cat #8961, Cell Signaling Technology). Fixative (200 mL of fresh 4% paraformaldehyde in 1x PBS) was pre-chilled to 4 °C. Groups of up to 8 slides with liver sections were taken directly from storage at − 20 °C and placed on a slide rack in pre-chilled fixative for 15 min at 4 °C. Slides were then removed from the fixative and dehydrated by immersion in 200 mL of 50% ethanol for 5 min, followed by 5 min in 70% ethanol, and then twice in 100% ethanol for 5 min. Slides were incubated at − 20 °C in fresh 100% ethanol overnight. At the start of the next day, a water bath and an Advanced Cell Diagnostics HybEZ oven were set to 40 °C after placing Advanced Cell Diagnostics humidity paper soaked in distilled water in the oven’s humidity control tray. Dehydrated slides were air dried on absorbent paper for 5 min at room temperature, and a hydrophobic barrier was drawn around each tissue slice using a special Advanced Cell Diagnostics marker pen and allowed to dry for 1 min. Slides were then placed on the HybEZ slide rack. Five drops of Pretreat 4 protease reagent was added to each section and then incubated for 30 min at room temperature with the incubation tray cover on. Materials for probe hybridization were prepared during this incubation step. 50x Wash Buffer (60 mL) was pre-warmed at 40 °C for 10–20 min before adding to 2.94 L of Milli-Q water in a sealable container to make 1x Wash Buffer (stable at room temperature for over 1 month). Probes were warmed at 40 °C for 10 min and cooled to room temperature before use, and the amplifying reagents (Amp1-FL to Amp4-FL) were warmed to room temperature. After 30 min, excess liquid was flicked off each slide and the slides were washed in 1x PBS twice by submerging the rack in the PBS wash 3–5 times. Slides were allowed to sit in 1x PBS for up to 15 min before proceeding to probe hybridization. Slides were then tapped gently to remove any excess liquid and placed on the HybEZ rack. Probe hybridization was performed using one of the following: mixture of ZZ probe sets for up to three RNAs of interest, each using a different fluorescent channel; RNAscope 3-plex Positive Control probes (Cat # 320881); or RNAscope 3-plex Negative Control Probe (Cat # 320871). Four drops of the above described probe mixture was added to each slide and the rack was placed in the oven for 2 h at 40 °C. After 2 h, the tray was removed from the oven, excess liquid was flicked from the slide, and the slide was washed twice in 1X wash buffer at room temperature for 2 min. This hybridization and washing procedure was repeated for each of four sequential amplification probes (Amp1-FL through Amp4-FL), with differing periods of incubation for each step: Amp1-FL for 30 min, Amp2-FL for 15 min, Amp3-FL for 30 min, and Amp4-FL for 15 min. We used the manufacturer’s Amp4 Alt A set-up (Cat. # 320855) for this experiment, which labels the Channel 1 probe with Alexa 488 (green) and the Channel 2 probe with Atto 550 (orange). Due to the high auto-fluorescence of liver tissue in the green spectrum, we visualized the most highly expressed RNAs in Channel 1 and the less highly expressed RNAs in Channel 2. Thus, for the sex-biased lncRNAs, *lnc7423* was visualized in Channel 1 and *lnc14770* in Channel 2; for the TCPOBOP-inducible genes, *Cyp2b10* was visualized in Channel 1 and *lnc5998* in Channel 2. After the final wash step, excess liquid was removed by gently tapping, and 20 μl of Prolong Gold Antifade with DAPI (Cat #8961, Cell Signaling Technology) was placed in the center of the slide. A coverslip was added and the slide stored in the dark at 4 °C.

Slides were imaged on a spinning disk confocal microscope (Olympus BX61) with a 60x oil immersion lens using preset channels to visualize DAPI (358 nm excitation/461 nm emission; blue), Alexa 488 (488/540 nm; green), Atto 550 (550/576 nm; orange) and Atto 647 (647/669 nm; far red). For processing, images were imported into FIJI image analysis software (https://imagej.net/Downloads) where channels were assigned to the proper visualization color and the z-stack was collapsed. Individual channels were separated and the delete background function was applied to each channel individually to remove excess noise, using a rolling ball radius of 50 pixels. Single channels were then merged to create a final image that was saved in RBG format to preserve the settings and converted to a .tif file. To quantify signal, the count dots feature of FIJI image analysis was applied to each channel individually, excluding DAPI, and counts were normalized over 5 fields of view for each measurement. Nuclei were counted manually for each image due to the variation of DAPI staining in the nucleus caused by euchromatin and heterochromatin.

### RNA-seq library preparation and sequencing

Sequencing libraries were prepared for 13 validated mouse livers (3 vehicle-treated males, 3 TCPOBOP-treated males, 3 vehicle-treated females, and 4 TCPOBOP-treated females) using RNA purified from each of the four subcellular fractions described above (cytoplasm, nucleus, nucleoplasm, chromatin-bound fraction). Illumina sequencing libraries were prepared using 0.5 μg of input RNA by poly(A) selection using the NEBNext Poly(A) mRNA Magnetic Isolation Module (Cat #E7490L), followed by library synthesis using the NEBNext Ultra Directional RNA Sequencing for Illumina kit (Cat #E7420L). An additional 0.5 μg of each of the 13 chromatin-bound (CB) RNA samples was also processed without poly(A) selection (CBnPAs fraction) to give a total of 13 livers × 5 fractions each = 65 RNA-seq libraries. Illumina sequencing was carried out by Novogene, Inc. (Sacramento, CA) and yielded a total of 1.68 billion 150 bp paired-end read sequence fragments. Raw and processed sequencing flies are available for download from GEO (https://www.ncbi.nlm.nih.gov/geo/) accession GSE160722. Counting and mapping statistics for each sequenced sample are found in Table S1A.

### Sequence read counting using custom GTF files

RNA-seq data was processed using a custom pipeline described elsewhere [32]. Briefly, sequence reads were mapped to the mouse genome (mm9) using TopHat (v2.1.1), FeatureCounts (1.4.6-p5) was used to count sequence read counts using custom gene transfer format (GTF) files, and EdgeR (exact test) was used to calculate differential gene expression and significance values. Adjusted *p*-values (i.e., false discovery rate) < 0.05 were considered significant; where indicated, more stringent thresholds for significance were applied. Custom GTF files used for read counting contained a total of 38,901 genes, based on the union of 15,558 lncRNA genes, whose full gene structures and isoforms/splice variants are reported elsewhere [26], and 24,197 RefSeq genes. To avoid duplicate counting, we removed a total of 854 RefSeq genes from the initial set of RefSeq genes, most of which are non-coding genes that substantially matched one of the 15,558 lncRNA structures, as follows. The extent of match between the set of 24,197 RefSeq genes and 15,558 lncRNA genes was initially determined using Bedtools intersect and Bedtools coverage commands, where > 30% overlap of a lncRNA structure with a non-coding RefSeq gene was deemed to be a significant match. Overlaps < 30% were then manually curated to identify RefSeq-lncRNA pairs with highly similar exonic features, as determined by visual assessment and best judgement. Based on these analyses, 608 RefSeq non-coding RNAs showed near perfect overlap (> 98% match) with our set of 15,558 lncRNA structures and were removed. A further 319 RefSeq genes (mostly non-coding genes) were 98% contained within a longer lncRNA structure, while 62 lncRNAs were > 98% within a longer RefSeq gene, most of which were protein-coding RNAs. 243 of the 319 RefSeq genes found within longer lncRNAs were determined to be the same as a lncRNA gene, based on the criteria described above, and 1 of the 62 RefSeq genes encompassing a shorter lncRNA was also considered to be the same as a lncRNA gene; those 244 RefSeq genes were also removed from the RefSeq list. Finally, two other RefSeq genes were lost due to Excel errors generating double entries for gene names converted to 1-Mar and 2-Mar (now renamed *Marchf1* and *Marchf2*) [106], leading to the final total of 38,901 RefSeq + lncRNA genes. These are listed in Table S1D, where the lncRNAs whose duplicate RefSeq entries were removed (608 identical matches plus 243 partially overlapping genes) are marked in column K.

Three separate GTF files were prepared for the set of 38,901 genes (Supplemental Files S1, S2, S3), respectively comprised of the following features for each gene: (1) Gene Body GTF, which includes the full genomic region of each gene, from the transcription start site to the transcript end site; (2) Exon Collapsed GTF, which includes all genomic regions that are exonic in any isoform of a gene; and (3) Intronic Only GTF, which includes all genomic sequences within intronic regions that are shared across all isoforms of a gene, i.e., regions do not overlap any exon in any isoform. Sequence reads were mapped using TopHat, and multi-mapped reads were removed from the BAM files, leaving only singly mapped reads, which were counted by featureCounts using the above custom GTF files. The MultiOverlap option of featureCounts was enabled by using the –O option, so that reads that overlap two or more genes in a GTF file were included in the counts for each gene. For many intragenic lncRNAs, the Gene Body counts and the Intronic Only counts were artificially high due to the inclusion of exonic reads from highly expressed, overlapping RefSeq protein-coding genes located within the lncRNA’s intronic regions. Similarly, many miRNAs are found within introns of highly expressed protein-coding genes, and consequently, their Gene Body counts are inflated due to the inclusion of spliced reads from the overlapping protein-coding genes. To mitigate these issues, the Gene Body and Intronic Only count files output by featureCounts were modified for all 249 intragenic lncRNA genes and all 1107 miRNA genes, as follows: Gene Body read counts were replaced by Exon Collapsed read counts, and Intronic Only read counts were set to zero. For those genes, the gene lengths used to calculate FPKM (fragments per kilobase length per million mapped sequence reads) values in downstream analyses were correspondingly modified to reflect the changes in counting regions for those genes. The relationship between Gene Body (GB) read counts and read counts mapping to Exon Collapsed (EC) regions plus those that map to Intronic Only (IO) regions is described in the legend to Table S1B-S1C and in data presented in those tables.

### Intronic/Exonic read density ratio

Genes with an intronic length of zero, i.e., all mono-exonic genes, were excluded from this analysis, as were all intragenic lncRNA and miRNA gene structures, due to the modifications to their sequence read counting described above. All other genes were separated into three categories: (1) Not Expressed, genes with an average across untreated male or female liver samples (*n* = 3 each) of < 3 reads per sample in EC regions and also in IO regions; (2) Low Expressed, genes that have an average maximum across untreated male or female samples of 3 to 9 reads per sample in either EC or IO regions; and (3) Expressed, genes with > 9 reads per sample, averaged across untreated male or female samples, in either EC or IO regions. A total of ~ 220–420 antisense lncRNAs, ~ 360–780 intergenic lncRNAs and 11,700–13,700 RefSeq genes met the criteria for Expressed in either male or female liver in each subcellular fraction (Table S1D, Table S1E). RefSeq gene accession numbers and gene names were used to classify genes as protein-coding (NM accession numbers only), non-coding (NR accession numbers only), protein-coding/non-coding (both NM and NR accession numbers are assigned to different isoforms of the same gene), snRNAs and miRNAs, and to remove genes with NR accession numbers, which reduced the overall list of 24,197 RefSeq genes to a list of 20,082 RefSeq PCGs. In total, 1442 multi-exonic lncRNA genes and 13,737 multi-exonic PCGs (Table S1F) were considered for this analysis across all 5 subcellular fractions, as presented in Fig. 2 for male liver and in Figure S2 for female liver; however, not all genes were expressed at a high enough level to be assessed for Intronic/Exonic read density in every fraction.

To calculate Intronic/Exonic read densities, we first added a pseudo-count of 0.1 reads to both the Exon Collapsed (EC) and Intronic Only (IO) read counts for each gene. EC and IO read counts for each gene were then normalized by the sequencing read depth of each sample, and the resulting normalized counts were averaged together across the n = 3 biological replicates for both counting regions. To compare sequence read density in intronic versus exonic regions, we first computed for each gene the fraction of the full length gene body that is in an IO region, and the fraction that is in an EC region. The mean normalized read counts for IO and for EC regions were then divided by their respective fraction of full gene body length to give a genomic length-weighted normalized average read count. For each gene, the intron/exon read density ratio was then determined by dividing the weighted normalized average read counts for the IO region by that of the EC region (Table S1F).

### Differential expression analysis: subcellular fraction bias

The pre-ribosomal RNA gene RNA45S was substantially removed during polyA selection and thus comprised only 0.2–14% of sequence reads in all except for the chromatin-bound non-PolyA-selected sequencing libraries, where it comprised 46–51% of all EC (exon collapsed) region reads; hence, RN45S sequence reads were removed from all 65 RNA-seq samples when analyzing differential expression between subcellular fractions. All samples were then normalized to 10 million EC region reads per sample, in order to compare the relative number of gene transcripts across the five subcellular fractions by differential expression analysis using edgeR (exact test). Differential expression comparisons were carried out between three pairs of subcellular fractions: (1) Cytoplasm vs Nucleus (both polyA-selected), (2) Nucleoplasm vs Chromatin Bound (both polyA-selected), and (3) Chromatin Bound (polyA-selected) vs Chromatin Bound (non-PolyA-selected). Each comparison was carried out for 4 different biological conditions: untreated male and female liver, and separately, 27-h TCPOBOP-stimulated male and female liver. Tables S2A-S2C present the differential expression results across all 4 biological conditions for each of the 3 pairs of subcellular fraction comparisons. For the final analysis of differentially expressed genes, only lncRNAs and PCGs were considered. We applied a stringent threshold for differential expression between subcellular fractions, edgeR-adjusted *p*-value < 0.001 in at least one of the four biological conditions, to identify genes showing a highly significant enrichment between subcellular fractions. Importantly, these analyses identified transcripts that show significant differential enrichment in the indicated subcellular fraction, but do not imply that a given transcript is exclusively present in that fraction. Where indicated, analyses considered genes showing enrichment between subcellular fractions at the relaxed significance of edgeR-adjusted *p*-value < 0.05. The magnitude of the subcellular fraction bias was taken as the normalized ratio of the FPKM expression values for each subcellular fraction, determined using exon collapsed reads, and is presented for the biological condition with the highest FPKM value that showed a significant bias.

### Discovery of sex-biased and TCPOBOP-responsive genes

Differential expression analysis was performed with edgeR (exact test) using the exon collapsed read counts and gene length GTF definitions for each of the five subcellular fractions examined. The percentage of sequence reads derived from RN45S was consistent within each subcellular fraction for all four biological conditions, and so RN45S sequence reads were not removed for these differential expression analyses. Comparisons were carried out between three sets of biological conditions: (1) Male vs Female liver, (2) Male vs 27 h TCPOBOP-treated Male liver, and (3) Female vs 27 h TCPOBOP-treated Female liver. These comparisons were performed separately using sequence reads from each of the five subcellular fractions: cytoplasm, nucleus, nucleoplasm, chromatin-bound (all polyA-selected) and chromatin-bound non-PolyA-selected. Data on the sex-bias and TCPOBOP-responsiveness of all 38,901 genes across the five fractions is shown in Table S3A and Table S3B, respectively. For these analyses, a gene was considered sex-biased or TCPOBOP-responsive if it met an edgeR-adjusted p-value cutoff of 0.05 for differential expression in at least one of the five fractions.

### Integration of prior datasets: liver sex-differences

Prior published RNA-seq datasets comparing gene expression in untreated male vs untreated female mouse liver were integrated and compared with the results obtained in this study (Table S4A, Table S4C, Table S4D). Differential sex-biased expression, and responsiveness to hypophysectomy [26] or to deletion of Ezh1/Ezh2 [107] were determined at a threshold of > 2-fold expression difference at an edgeR-adjusted *p*-value (FDR) < 0.05. The prior RNA-seq datasets used in this analysis used either total, nuclear or cytoplasmic liver RNA, with either polyA selection or Ribo-minus ribosomal RNA depletion, and either CD-1 mouse livers [23, 32, 34, 47] or C57BL/6 J mouse livers [108], as indicated in Table S4. Antisense lncRNAs were not considered for the datasets obtained by unstranded RNA sequencing, as the genomic strand of the sequence reads could not be determined. RNA-seq datasets comparing liver expression in intact male or intact female mice to that in hypophysectomized male or female mice were included to determine the response of sex-biased lncRNAs to loss of pituitary-dependent growth hormone signaling. Class 1 lncRNAs are those that, following hypophysectomy, show decreased expression in the sex where the lncRNA is more highly expressed in intact mouse liver. In contrast, Class 2 lncRNAs increase in expression following hypophysectomy in the sex where they show lower expression in intact mice [26]. Hypophysectomy class assignments were made for lncRNAs that showed sex-biased expression in at least one of the five subcellular fractions in this study, or in at least one of the prior datasets described above (Table S4A, column L). Sex-biased lncRNAs whose expression levels significantly change in male liver after 20 days of age, as we determined elsewhere [26], were assigned to four classes (Table S4A, column M). Interesting sets of robust, novel and non-responsive sex-biased lncRNAs (Table S4A, column J) are identified in Table S4B.

### Integration of prior datasets: TCPOBOP-responsiveness

The current analysis of genes showing TCPOBOP responsiveness in one or more subcellular fractions (Table S3B) was integrated with five prior datasets examining responsiveness to TCPOBOP (Table S5C): four polyA-selected nuclear RNA datasets from 3-h and 27-h TCPOBOP-exposed Male and Female liver vs sex-matched vehicle controls (series G123), and one polyA-selected total RNA dataset from 3-h TCPOBOP-treated Male vs vehicle-treated Male liver (series G95) [22]. LncRNAs were identified as significantly up regulated or significantly down regulated by TCPOBOP or other xenobiotic exposures (Table S5C) using a threshold of normalized absolute fold-change > 2 and an edgeR-adjusted p-value < 0.05. If datasets conflicted, e.g., where a gene was UP in one data set and DOWN in another, the response was characterized as MIXED. Other xenobiotic exposures examined (Table S5C) include: phenobarbital treatment of male liver (GSE77729), acetaminophen exposure of male liver (GSE111828), TCPOBOP or CITGO exposure of livers of mouse CAR mice or human CAR transgenic mice (GSE98666), and WY14634 or fenofibrate treatment of both mouse PPAR mice and human PPAR transgenic mice (GSE132386; series G134). Results are integrated in Table S5A, and descriptions of interesting groups of robust, novel and non-responding TCPOBOP-responsive lncRNAs (Table S5A, column J) are presented in Table S5B.

### Analysis of lncRNAs: proximity and genomic organization

The RefSeq gene closest in linear distance to the gene body of each lncRNA gene, without regard to direction or genomic strand, was determined using the bedtools closest function. RefSeq genes that overlap a lncRNA gene on either strand were assigned a distance of zero. If multiple genes were discovered at the same distance, all were considered in this analysis. Divergently transcribed RefSeq genes met all three of these criteria: transcription start site (TSS) within 5 kb of a lncRNA TSS; does not overlap the lncRNA gene; and is transcribed from the opposite strand. Genes that overlap a lncRNA gene were characterized as antisense if they are on the opposite strand; they were designated intragenic if they are on the same strand. The union of the Closest, Antisense, Intergenic and Divergent RefSeq gene lists was compared to six lists of RefSeq genes relating to liver function and disease: 326 sex-biased RefSeq genes (padj < 0.05) and 1815 TCPOBOP-responsive RefSeq genes (padj < 0.05), both from this study across any subcellular fraction; and 772 non-alcoholic steatohepatitis-responsive genes based on single cell RNA-seq analysis [109], 107 non-alcoholic fatty liver disease-related genes [110], 217 liver-related fibrosis genes [107], and 920 hepatocellular carcinoma-related genes [107]. These gene lists are integrated in Table S4A and Table S5A (last columns). RefSeq genes that are within the same topologically associated domain (TAD) as lncRNAs, and that show sex-biased expression or TCPOBOP responsiveness in one or more subcellular fraction, are shown in Table S6. Determination of TAD regions, including inter-TAD and imputed TAD region boundaries, was based on analyses performed by Gracia Bonilla of this laboratory, and was computed based on 3538 TAD regions obtained from Hi-C data [57, 111] and 2403 inter-TAD regions predicted computationally in male mouse liver [57] (Table S6C).

### Graphing and statistics

Error bars shown in scatter dot plot column graphs represent the inter-quartile range (IQR) of the distribution from the median. Statistical analysis for these graphs was performed using the Kruskal-Wasllis test followed by Dunn’s multiple comparison tests to obtain adjusted *p*-values, as implemented in GraphPad Prism software to compare the distributions of multiple unmatched groups in a nonparametric manner.

## Supplementary Information

**Additional file 1. Table S1.**

**Additional file 2. Table S2.**

**Additional file 3. Table S3.**

**Additional file 4. Table S4.**

**Additional file 5. Table S5.**

**Additional file 6. Table S6.**

**Additional file 7. Table S7.**

**Additional file 8. Supplemental Figures S1-S6.**

**Additional file 9.** File S1. Gene Body GTF.

**Additional file 10.** File S2. Exon Collapsed GTF.

**Additional file 11.** File S3. Intronic Only GTF.

## Data Availability

The datasets generated and/or analyzed during the current study are included in this published article and its supplementary information files. Raw and processed sequencing files are available from GEO (https://www.ncbi.nlm.nih.gov/geo/) accession GSE160722.

## Abbreviations

CAR: constitutive androstane receptor
CBnPAs: chromatin-bound, non-polyA-selected
DHS: DNase hypersensitive site(s)
EC: exon collapsed
FDR: false discovery rate
FPKM: fragments per kilobase length per million mapped sequence reads
GB: gene body
GTF: gene transfer format
IO: intronic only
IQR: inter-quartile range
lncRNA: long non-coding RNA
PCGs: protein coding genes
smFISH: single molecule RNA fluorescence in situ hybridization
TAD: topologically associated domain
TCPOBOP: 1,4-bis (2-(3,5-dichloropyridyloxy))benzene)
TSS: transcription start site

## References

[1] Guttman M, Amit I, Garber M, French C, Lin MF, Feldser D, Huarte M, Zuk O, Carey BW, Cassady JP (2009). Chromatin signature reveals over a thousand highly conserved large non-coding RNAs in mammals. Nature.

[2] Mahpour A, Mullen AC (2021). Our emerging understanding of the roles of long non-coding RNAs in normal liver function, disease, and malignancy. JHEP reports : innovation in hepatology.

[3] Peng H, Wan LY, Liang JJ, Zhang YQ, Ai WB, Wu JF (2018). The roles of lncRNA in hepatic fibrosis. Cell & bioscience.

[4] Wong CM, Tsang FH, Ng IO (2018). Non-coding RNAs in hepatocellular carcinoma: molecular functions and pathological implications. Nat Rev Gastroenterol Hepatol.

[5] De Santa F, Barozzi I, Mietton F, Ghisletti S, Polletti S, Tusi BK, Muller H, Ragoussis J, Wei CL, Natoli G (2010). A large fraction of extragenic RNA pol II transcription sites overlap enhancers. PLoS Biol.

[6] Ransohoff JD, Wei Y, Khavari PA (2018). The functions and unique features of long intergenic non-coding RNA. Nat Rev Mol Cell Biol.

[7] Yao RW, Wang Y, Chen LL (2019). Cellular functions of long noncoding RNAs. Nat Cell Biol.

[8] Kopp F, Mendell JT (2018). Functional classification and experimental dissection of long noncoding RNAs. Cell.

[9] Zhang X, Wang W, Zhu W, Dong J, Cheng Y, Yin Z, Shen F (2019). Mechanisms and Functions of Long Non-Coding RNAs at Multiple Regulatory Levels. Int J Mol Sci.

[10] Loda A, Heard E (2019). Xist RNA in action: past, present, and future. PLoS Genet.

[11] Sahakyan A, Yang Y, Plath K (2018). The role of Xist in X-chromosome dosage compensation. Trends Cell Biol.

[12] Rajagopal T, Talluri S, Akshaya RL, Dunna NR (2020). HOTAIR LncRNA: A novel oncogenic propellant in human cancer. Clinica chimica acta.

[13] Pradas-Juni M, Hansmeier NR, Link JC, Schmidt E, Larsen BD, Klemm P, Meola N, Topel H, Loureiro R, Dhaouadi I (2020). A MAFG-lncRNA axis links systemic nutrient abundance to hepatic glucose metabolism. Nat Commun.

[14] Song Y, Liu C, Liu X, Trottier J, Beaudoin M, Zhang L, Pope C, Peng G, Barbier O, Zhong X (2017). H19 promotes cholestatic liver fibrosis by preventing ZEB1-mediated inhibition of epithelial cell adhesion molecule. Hepatology.

[15] He Y, Wu YT, Huang C, Meng XM, Ma TT, Wu BM, Xu FY, Zhang L, Lv XW, Li J (2014). Inhibitory effects of long noncoding RNA MEG3 on hepatic stellate cells activation and liver fibrogenesis. Biochim Biophys Acta.

[16] Li Z, Wang J, Zeng Q, Hu C, Zhang J, Wang H, Yan J, Li H, Yu Z (2018). Long noncoding RNA HOTTIP promotes mouse hepatic stellate cell activation via Downregulating miR-148a. Cell Physiol Biochem.

[17] Dunagin M, Cabili MN, Rinn J, Raj A (2015). Visualization of lncRNA by single-molecule fluorescence in situ hybridization. Methods Mol Biol.

[18] Cabili MN, Dunagin MC, McClanahan PD, Biaesch A, Padovan-Merhar O, Regev A, Rinn JL, Raj A (2015). Localization and abundance analysis of human lncRNAs at single-cell and single-molecule resolution. Genome Biol.

[19] Chen KH, Boettiger AN, Moffitt JR, Wang S, Zhuang X (2015). RNA imaging. Spatially resolved, highly multiplexed RNA profiling in single cells. Science.

[20] Moffitt JR, Hao J, Wang G, Chen KH, Babcock HP, Zhuang X (2016). High-throughput single-cell gene-expression profiling with multiplexed error-robust fluorescence in situ hybridization. Proc Natl Acad Sci U S A.

[21] Carlevaro-Fita J, Johnson R (2019). Global positioning system: understanding long noncoding RNAs through subcellular localization. Mol Cell.

[22] Lodato NJ, Melia T, Rampersaud A, Waxman DJ (2017). Sex-differential responses of tumor promotion-associated genes and Dysregulation of novel long noncoding RNAs in constitutive Androstane receptor-activated mouse liver. Toxicological Sci.

[23] Melia T, Hao P, Yilmaz F, Waxman DJ (2016). Hepatic long Intergenic noncoding RNAs: high promoter conservation and dynamic, sex-dependent transcriptional regulation by growth hormone. Mol Cell Biol.

[24] Chow EY, Zhang J, Qin H, Chan TF (2019). Characterization of hepatocellular carcinoma cell lines using a fractionation-then-sequencing approach reveals nuclear-enriched HCC-associated lncRNAs. Front Genet.

[25] Noh JH, Kim KM, McClusky WG, Abdelmohsen K, Gorospe M (2018). Cytoplasmic functions of long noncoding RNAs. Wiley Interdiscip Rev RNA.

[26] Melia T, Waxman DJ (2019). Sex-biased lncRNAs inversely correlate with sex-opposite gene Coexpression networks in diversity outbred mouse liver. Endocrinology.

[27] Karri K, Waxman DJ (2020). Widespread dysregulation of long noncoding genes associated with fatty acid metabolism, cell division, and immune response gene networks in xenobiotic-exposed rat liver. Toxicological Sci.

[28] Davidovich C, Wang X, Cifuentes-Rojas C, Goodrich KJ, Gooding AR, Lee JT, Cech TR (2015). Toward a consensus on the binding specificity and promiscuity of PRC2 for RNA. Mol Cell.

[29] Chu C, Qu K, Zhong FL, Artandi SE, Chang HY (2011). Genomic maps of long noncoding RNA occupancy reveal principles of RNA-chromatin interactions. Mol Cell.

[30] Bhatt DM, Pandya-Jones A, Tong AJ, Barozzi I, Lissner MM, Natoli G, Black DL, Smale ST (2012). Transcript dynamics of proinflammatory genes revealed by sequence analysis of subcellular RNA fractions. Cell.

[31] Werner MS, Ruthenburg AJ (2015). Nuclear fractionation reveals thousands of chromatin-tethered noncoding RNAs adjacent to active genes. Cell Rep.

[32] Connerney J, Lau-Corona D, Rampersaud A, Waxman DJ (2017). Activation of male liver chromatin accessibility and STAT5-dependent gene transcription by plasma growth hormone pulses. Endocrinology.

[33] Hao P, Waxman DJ (2021). STAT5 Regulation of Sex-Dependent Hepatic CpG Methylation at Distal Regulatory Elements Mapping to Sex-Biased Genes. Mol Cell Biol.

[34] Lau-Corona D, Suvorov A, Waxman DJ (2017). Feminization of Male Mouse Liver by Persistent Growth Hormone Stimulation: Activation of Sex-Biased Transcriptional Networks and Dynamic Changes in Chromatin States. Mol Cell Biol.

[35] Melia T, Waxman DJ (2020). Genetic factors contributing to extensive variability of sex-specific hepatic gene expression in diversity outbred mice. PLoS One.

[36] Brocker CN, Kim D, Melia T, Karri K, Velenosi TJ, Takahashi S, Aibara D, Bonzo JA, Levi M, Waxman DJ (2020). Long non-coding RNA Gm15441 attenuates hepatic inflammasome activation in response to PPARA agonism and fasting. Nat Commun.

[37] Dempsey JL, Cui JY (2019). Regulation of hepatic long noncoding RNAs by Pregnane X receptor and constitutive Androstane receptor agonists in mouse liver. Drug Metab Dis.

[38] Pouché L, Vitobello A, Römer M, Glogovac M, MacLeod AK, Ellinger-Ziegelbauer H, Westphal M, Dubost V, Stiehl DP, Dumotier B (2017). Xenobiotic CAR activators induce Dlk1-Dio3 locus noncoding RNA expression in mouse liver. Toxicological Sci.

[39] Chen L, Wang P, Manautou JE, Zhong XB (2020). Knockdown of long noncoding RNAs hepatocyte nuclear factor 1α antisense RNA 1 and hepatocyte nuclear factor 4α antisense RNA 1 alters susceptibility of acetaminophen-induced cytotoxicity in HepaRG cells. Mol Pharmacol.

[40] Tzameli I, Pissios P, Schuetz EG, Moore DD (2000). The xenobiotic compound 1,4-bis [2-(3,5-dichloropyridyloxy)]benzene is an agonist ligand for the nuclear receptor CAR. Mol Cell Biol.

[41] Cheng SL, Bammler TK, Cui JY (2017). RNA sequencing reveals age and species differences of constitutive Androstane receptor-targeted drug-processing genes in the liver. Drug Metab Disposition.

[42] Ling G, Sugathan A, Mazor T, Fraenkel E, Waxman DJ (2010). Unbiased, genome-wide in vivo mapping of transcriptional regulatory elements reveals sex differences in chromatin structure associated with sex-specific liver gene expression. Mol Cell Biol.

[43] Stewart M (2019). Polyadenylation and nuclear export of mRNAs. J Biol Chem.

[44] Tilgner H, Knowles DG, Johnson R, Davis CA, Chakrabortty S, Djebali S, Curado J, Snyder M, Gingeras TR, Guigo R (2012). Deep sequencing of subcellular RNA fractions shows splicing to be predominantly co-transcriptional in the human genome but inefficient for lncRNAs. Genome Res.

[45] Marzluff WF, Koreski KP (2017). Birth and death of histone mRNAs. Trends in Genetics : TIG.

[46] Wang F, Flanagan J, Su N, Wang LC, Bui S, Nielson A, Wu X, Vo HT, Ma XJ, Luo Y (2012). RNAscope: a novel in situ RNA analysis platform for formalin-fixed, paraffin-embedded tissues. J Mol Diagn.

[47] Sugathan A, Waxman DJ (2013). Genome-wide analysis of chromatin states reveals distinct mechanisms of sex-dependent gene regulation in male and female mouse liver. Mol Cell Biol.

[48] Laz EV, Holloway MG, Chen CS, Waxman DJ. Characterization of three growth hormone-responsive transcription factors preferentially expressed in adult female liver. Endocrinology 2007, 148(7):3327-3337. 10.1210/en.2006-1192 PMC2585771 17412818

[49] Conforto TL, Waxman DJ (2012). Sex-specific mouse liver gene expression: genome-wide analysis of developmental changes from pre-pubertal period to young adulthood. Biol Sex Differ.

[50] Wang J, Pu J, Zhang Y, Yao T, Luo Z, Li W, Xu G, Liu J, Wei W, Deng Y (2019). DANCR contributed to hepatocellular carcinoma malignancy via sponging miR-216a-5p and modulating KLF12. J Cell Physiol.

[51] Qu A, Yang Q (2020). LncRNA SNHG1 promotes cell progression and metastasis via sponging miR-377-3p in hepatocellular carcinoma. Neoplasma.

[52] Lan T, Yuan K, Yan X, Xu L, Liao H, Hao X, Wang J, Liu H, Chen X, Xie K (2019). LncRNA SNHG10 facilitates Hepatocarcinogenesis and metastasis by modulating its homolog SCARNA13 via a positive feedback loop. Cancer Res.

[53] He Z, Yang D, Fan X, Zhang M, Li Y, Gu X, Yang M (2020). The Roles and Mechanisms of lncRNAs in Liver Fibrosis. Int J Mol Sci.

[54] Engreitz JM, Haines JE, Perez EM, Munson G, Chen J, Kane M, McDonel PE, Guttman M, Lander ES (2016). Local regulation of gene expression by lncRNA promoters, transcription and splicing. Nature.

[55] Gil N, Ulitsky I (2020). Regulation of gene expression by cis-acting long non-coding RNAs. Nat Rev Genet.

[56] Sikorska N, Sexton T (2020). Defining functionally relevant spatial chromatin domains: it is a TAD complicated. J Mol Biol.

[57] Matthews BJ, Waxman DJ. Computational prediction of CTCF/cohesin-based intra-TAD loops that insulate chromatin contacts and gene expression in mouse liver. Elife. 2018;7. 10.7554/eLife.34077 PMC5986275 29757144

[58] Matthews BJ, Waxman DJ (2020). Impact of 3D genome organization, guided by cohesin and CTCF looping, on sex-biased chromatin interactions and gene expression in mouse liver. Epigenetics Chromatin.

[59] Luo S, Lu JY, Liu L, Yin Y, Chen C, Han X, Wu B, Xu R, Liu W, Yan P (2016). Divergent lncRNAs regulate gene expression and lineage differentiation in pluripotent cells. Cell Stem Cell.

[60] Ram PA, Waxman DJ (1999). SOCS/CIS protein inhibition of growth hormone-stimulated STAT5 signaling by multiple mechanisms. J Biol Chem.

[61] Greenhalgh CJ, Rico-Bautista E, Lorentzon M, Thaus AL, Morgan PO, Willson TA, Zervoudakis P, Metcalf D, Street I, Nicola NA (2005). SOCS2 negatively regulates growth hormone action in vitro and in vivo. J Clin Invest.

[62] Ram PA, Waxman DJ (2000). Role of the cytokine-inducible SH2 protein CIS in desensitization of STAT5b signaling by continuous growth hormone. J Biol Chem.

[63] Cui M, Sun J, Hou J, Fang T, Wang X, Ge C, Zhao F, Chen T, Xie H, Cui Y (2016). The suppressor of cytokine signaling 2 (SOCS2) inhibits tumor metastasis in hepatocellular carcinoma. Tumour Biol.

[64] Petrick JL, Braunlin M, Laversanne M, Valery PC, Bray F, McGlynn KA (2016). International trends in liver cancer incidence, overall and by histologic subtype, 1978-2007. Int J Cancer.

[65] Wands J (2007). Hepatocellular carcinoma and sex. N Engl J Med.

[66] Zhang Y, Laz EV, Waxman DJ (2012). Dynamic, sex-differential STAT5 and BCL6 binding to sex-biased, growth hormone-regulated genes in adult mouse liver. Mol Cell Biol.

[67] Mullican SE, Rangwala SM (2018). Uniting GDF15 and GFRAL: therapeutic opportunities in obesity and beyond. Trends Endocrinol Metab.

[68] Li D, Zhang H, Zhong Y (2018). Hepatic GDF15 is regulated by CHOP of the unfolded protein response and alleviates NAFLD progression in obese mice. Biochem Biophys Res Commun.

[69] Zhang M, Sun W, Qian J, Tang Y (2018). Fasting exacerbates hepatic growth differentiation factor 15 to promote fatty acid beta-oxidation and ketogenesis via activating XBP1 signaling in liver. Redox Biol.

[70] Tran T, Yang J, Gardner J, Xiong Y (2018). GDF15 deficiency promotes high fat diet-induced obesity in mice. PLoS One.

[71] Chung HK, Kim JT, Kim HW, Kwon M, Kim SY, Shong M, Kim KS, Yi HS (2017). GDF15 deficiency exacerbates chronic alcohol- and carbon tetrachloride-induced liver injury. Sci Rep.

[72] Conforto TL, Steinhardt GF, Waxman DJ (2015). Cross talk between GH-regulated transcription factors HNF6 and CUX2 in adult mouse liver. Mol Endocrinol.

[73] Higuchi C, Nakatsuka A, Eguchi J, Teshigawara S, Kanzaki M, Katayama A, Yamaguchi S, Takahashi N, Murakami K, Ogawa D (2015). Identification of circulating miR-101, miR-375 and miR-802 as biomarkers for type 2 diabetes. Metabolism.

[74] Kornfeld JW, Baitzel C, Konner AC, Nicholls HT, Vogt MC, Herrmanns K, Scheja L, Haumaitre C, Wolf AM, Knippschild U (2013). Obesity-induced overexpression of miR-802 impairs glucose metabolism through silencing of Hnf1b. Nature.

[75] Yang X, Xing H, Liu J, Yang L, Ma H, Ma H (2019). MicroRNA802 increases hepatic oxidative stress and induces insulin resistance in highfat fed mice. Mol Med Rep.

[76] Zhang C, Seo J, Murakami K, Salem ESB, Bernhard E, Borra VJ, Choi K, Yuan CL, Chan CC, Chen X (2018). Hepatic Ago2-mediated RNA silencing controls energy metabolism linked to AMPK activation and obesity-associated pathophysiology. Nat Commun.

[77] Hao P, Waxman DJ (2018). Functional roles of sex-biased, growth hormone-regulated MicroRNAs miR-1948 and miR-802 in young adult mouse liver. Endocrinology.

[78] Wang S, Hou Y, Chen W, Wang J, Xie W, Zhang X, Zeng L (2018). KIF9AS1, LINC01272 and DIO3OS lncRNAs AS novel biomarkers for inflammatory bowel disease. Mol Med Rep.

[79] Hernandez A, Garcia B, Obregon MJ (2007). Gene expression from the imprinted Dio3 locus is associated with cell proliferation of cultured brown adipocytes. Endocrinology.

[80] Hernandez A (2005). Structure and function of the type 3 deiodinase gene. Thyroid.

[81] Qatanani M, Zhang J, Moore DD (2005). Role of the constitutive androstane receptor in xenobiotic-induced thyroid hormone metabolism. Endocrinology.

[82] Maglich JM, Watson J, McMillen PJ, Goodwin B, Willson TM, Moore JT (2004). The nuclear receptor CAR is a regulator of thyroid hormone metabolism during caloric restriction. J Biol Chem.

[83] Rinn JL, Chang HY (2020). Long noncoding RNAs: molecular modalities to organismal functions. Annu Rev Biochem.

[84] Brugiolo M, Herzel L, Neugebauer KM: Counting on co-transcriptional splicing. *F1000Prime Rep* 2013, 5:9. 10.12703/P5-9 PMC3619158 23638305

[85] Guenzl PM, Barlow DP (2012). Macro lncRNAs: a new layer of cis-regulatory information in the mammalian genome. RNA Biol.

[86] Middleton R, Gao D, Thomas A, Singh B, Au A, Wong JJ, Bomane A, Cosson B, Eyras E, Rasko JE (2017). IRFinder: assessing the impact of intron retention on mammalian gene expression. Genome Biol.

[87] Palazzo AF, Koonin EV (2020). Functional long non-coding RNAs evolve from junk transcripts. Cell.

[88] Zuckerman B, Ulitsky I (2019). Predictive models of subcellular localization of long RNAs. RNA.

[89] Guo CJ, Xu G, Chen LL (2020). Mechanisms of long noncoding RNA nuclear retention. Trends Biochem Sci.

[90] Palazzo AF, Lee ES (2018). Sequence determinants for nuclear retention and cytoplasmic export of mRNAs and lncRNAs. Front Genet.

[91] Yin Y, Lu JY, Zhang X, Shao W, Xu Y, Li P, Hong Y, Cui L, Shan G, Tian B (2020). U1 snRNP regulates chromatin retention of noncoding RNAs. Nature.

[92] Jarroux J, Morillon A, Pinskaya M (2017). History, discovery, and classification of lncRNAs. Adv Exp Med Biol.

[93] Gudenas BL, Wang L (2018). Prediction of LncRNA subcellular localization with deep learning from sequence features. Sci Rep.

[94] Wegener M, Muller-McNicoll M (2018). Nuclear retention of mRNAs - quality control, gene regulation and human disease. Semin Cell Dev Biol.

[95] Jacob AG, Smith CWJ (2017). Intron retention as a component of regulated gene expression programs. Hum Genet.

[96] Boutz PL, Bhutkar A, Sharp PA (2015). Detained introns are a novel, widespread class of post-transcriptionally spliced introns. Genes Dev.

[97] Prasanth KV, Prasanth SG, Xuan Z, Hearn S, Freier SM, Bennett CF, Zhang MQ, Spector DL (2005). Regulating gene expression through RNA nuclear retention. Cell.

[98] Bahar Halpern K, Caspi I, Lemze D, Levy M, Landen S, Elinav E, Ulitsky I, Itzkovitz S (2015). Nuclear retention of mRNA in mammalian tissues. Cell Rep.

[99] Gao J, He J, Zhai Y, Wada T, Xie W (2009). The constitutive androstane receptor is an anti-obesity nuclear receptor that improves insulin sensitivity. J Biol Chem.

[100] Maglich JM, Lobe DC, Moore JT (2009). The nuclear receptor CAR (NR1I3) regulates serum triglyceride levels under conditions of metabolic stress. J Lipid Res.

[101] Thomson DW, Dinger ME (2016). Endogenous microRNA sponges: evidence and controversy. Nat Rev Genet.

[102] Paraskevopoulou MD, Hatzigeorgiou AG (2016). Analyzing MiRNA-LncRNA interactions. Methods Mol Biol.

[103] Paraskevopoulou MD, Vlachos IS, Karagkouni D, Georgakilas G, Kanellos I, Vergoulis T, Zagganas K, Tsanakas P, Floros E, Dalamagas T (2016). DIANA-LncBase v2: indexing microRNA targets on non-coding transcripts. Nucleic Acids Res.

[104] Percie du Sert N, Hurst V, Ahluwalia A, Alam S, Avey MT, Baker M, Browne WJ, Clark A, Cuthill IC, Dirnagl U *et al*: The ARRIVE guidelines 2.0: Updated guidelines for reporting animal research. *PLoS biology* 2020, 18(7):e3000410. 10.1371/journal.pbio.3000410 PMC7360023 32663219

[105] Hirabayashi S, Bhagat S, Matsuki Y, Takegami Y, Uehata T, Kanemaru A, Itoh M, Shirakawa K, Takaori-Kondo A, Takeuchi O (2019). NET-CAGE characterizes the dynamics and topology of human transcribed cis-regulatory elements. Nat Genet.

[106] Bruford EA, Braschi B, Denny P, Jones TEM, Seal RL, Tweedie S (2020). Guidelines for human gene nomenclature. Nat Genet.

[107] Lau-Corona D, Bae WK, Hennighausen L, Waxman DJ (2020). Sex-biased genetic programs in liver metabolism and liver fibrosis are controlled by EZH1 and EZH2. PLoS Genet.

[108] Lowe R, Gemma C, Rakyan VK, Holland ML (2015). Sexually dimorphic gene expression emerges with embryonic genome activation and is dynamic throughout development. BMC Genomics.

[109] Xiong X, Kuang H, Ansari S, Liu T, Gong J, Wang S, Zhao XY, Ji Y, Li C, Guo L (2019). Landscape of intercellular crosstalk in healthy and NASH liver revealed by single-cell Secretome gene analysis. Mol Cell.

[110] Chella Krishnan K, Kurt Z, Barrere-Cain R, Sabir S, Das A, Floyd R, Vergnes L, Zhao Y, Che N, Charugundla S (2018). Integration of multi-omics data from mouse diversity panel highlights mitochondrial dysfunction in non-alcoholic fatty liver disease. Cell Syst.

[111] Vietri Rudan M, Barrington C, Henderson S, Ernst C, Odom DT, Tanay A, Hadjur S (2015). Comparative hi-C reveals that CTCF underlies evolution of chromosomal domain architecture. Cell Rep.

