# Supplementary material for: Global analysis of expression, maturation and subcellular localization of mouse liver transcriptome identifies novel sex-biased and TCPOBOP-responsive long non-coding RNAs
Source: BMC Genomics. 2021 Mar 24;22:212. doi: 10.1186/s12864-021-07478-5 (PMC7992343; doi:10.1186/s12864-021-07478-5)
Supplement: Supplementary file 8 — Additional file 8. Supplemental Figures S1-S6. [file 12864_2021_7478_MOESM8_ESM.pdf]

Supplementary Figures for:

Global analysis of expression, maturation and subcellular localization of mouse liver transcriptome identifies novel sex-biased and TCPOBOP-responsive long non-coding RNAs

Christine N. Goldfarb and David J. Waxman

Department of Biology and Bioinformatics Program

Boston University, Boston, MA 02215

Additional Files

Additional File Fig. S1. Schematic outline of the purification of mouse liver RNA from liver subcellular fractions - From left to right, liver tissue frozen at -80C was homogenized as described in Methods then centrifuged to separate nuclei from the cellular lysate, which were respectively used to purify cytoplasmic and nuclear RNA. Nuclei were extracted with urea and high salt to obtain a nuclear lysate and chromatin pellet, which were used to purify the corresponding two RNA fractions. RNA isolated from each of the four subcellular fractions (cytoplasm, nucleus, nucleoplasm, chromatin-bound) was polyA-selected and Illumina RNA-seq libraries then prepared. Additionally, a non-polyA-selected RNA-seq Library was prepared from the chromatin-bound fraction. Differential expression analysis was performed using edgeR for the three pairwise comparisons shown at the right: cytoplasm vs nucleus, nucleoplasm vs chromatin-bound, and chromatin-bound (polyA-selected) vs chromatin-bound (non-polyA-selected) to identify lncRNA and PCG transcripts significantly enriched in each subcellular fraction.

Additional File Fig. S2. Transcript maturity across subcellular fractions by analysis of IO/EC read density ratios. The distribution of IO/EC read density ratios for individual genes, calculated by dividing the weighted normalized read density of the intronic only (IO) reads vs the exonic collapsed (EC) reads for 1,442 multi-exonic lncRNAs and 13,737 multi-exonic PCGs expressed in vehicle treated female liver in any fraction (mean of n=3 livers). The number of genes expressed per subcellular fraction is shown under each column: Cytoplasm (Cyto), Nucleus (Nuc), Nucleoplasm (NP), Chromatin-bound (CB), Chromatin- bound non-PolyA selected (CBnPAs). Error bars represent the interquartile range of the distribution from the median (horizontal midline). Black brackets compare lncRNA to PCG ratios within the same fraction and red brackets compare lncRNA ratios, or PCG ratios, between fractions (** = adjusted p-value < 0.0001). See Fig. 2 for corresponding results in vehicle-treated male liver. The underlying data used to generate these graphs are found in Table S1F. (**B**) and (**C**) UCSC Browser screen shot showing BigWig files of minus strand sequence reads for each of the five indicated subcellular fractions for Xist (lnc15394) and Cux2 in untreated female mouse liver. Extensive reads seen across the gene body in the chromatin bound fraction are substantially depleted after polyA-selection (top vs second reads track); however, multiple distinct peaks within intronic regions remain, most notably for Cux2. BigWig Y-axis scales are marked on the left. Both genes show female-specific

expression, with many fewer sequence reads in corresponding fractions from male liver (not shown). DHS, DNase hypersensitivity sites, indicating open chromatin. These same patterns were seen in all three biological replicates. DHS showing significantly greater accessibility in female liver are marked in pink, and one male-biased DHS within an intron of Cux2 is marked in blue [101]. Also see Fig. 2.

Additional File Fig. S3. Expression, fold-change and gene length/percent intronic length data for cytoplasmic versus nuclear fractions. Log2 fold-change versus log10 FPKM of cytoplasmic (positive y-axis values) versus nuclear lncRNAs (negative y-axis values) (A) and PCGs (B). Data shown is the same as in Fig. 3A, but presented here as separate graphs for lncRNAs and PCGs to facilitate their visual comparison (also see Table S2A, columns D and E). Comparison of the cytoplasmic biased (Cyto), unbiased (UB) and nuclear biased (Nuc) gene lengths (C) and % intronic length (D) (Table S1D, columns M-Q). A significant difference between the Cyto-biased and Nuc-biased genes was seen for PCGs, but not for lncRNAs, but only in the gene length comparisons. Error bars represent the interquartile range of the distribution from the median (horizontal midline). Red brackets compare lncRNA ratios, or PCG ratios, between sex-biased groups (** = adjusted p-value < 0.0001).

Additional File Fig. S4. Expression, fold-change, transcript maturity and gene length/percent intronic length for nucleoplasmic versus chromatin-bound fractions. (**A**) Log2 fold-change versus log10 FPKM of nucleoplasmic (positive y-axis values) versus chromatin-bound (negative y-axis values) for lncRNAs (left) and PCGs (right). Data shown is the same as in Fig. S5A, but presented here as separate graphs for lncRNAs and PCGs to facilitate their visual comparison (also see Table S2B, columns D and E). (**B**) Shown are log2 fold-change versus log10 FPKM values, as in A, for 7,719 other lncRNAs, all expressed at a maximum FPKM > 0.1, but which did not meet our stringent criteria of adjusted p-value < 0.001 for chromatin or nucleoplasmic bias. 7,095 (92%) of these lncRNAs were more abundant in the chromatin-bound fraction (Table S2B). In many cases, the bias of these lncRNAs for the chromatin-bound fraction was very strong (y-axis values down to log2 fold-change < -4) but missed our stringent significance threshold owing to their low expression (~80% at FPKM < 1). (**C**) Strength of compartment bias [median chromatin/nucleoplasm ratio: PCGs, 3.1 (IQR, 2.5 to 4.5), lncRNAs, 18.5 (IQR, 8.4 to 60.4] and (**D**) distributions of FPKM expression levels for lncRNAs and PCGs, grouped based on their nucleoplasmic (NP) or chromatin-bound fraction bias (CB). (**E**) Transcript maturity (IO/EC read density ratio, Table S1F) is graphed for the nucleoplasmic and chromatin-bound compartments for the lncRNAs and PCGs that show significant nucleoplasmic bias (NP) or chromatin-bound fraction bias (CB), or are unbiased (UB) with regards to these two compartments. Only multi-exonic genes were included in this analysis. There was no significant difference between NP, UB or CB lncRNAs between the NP and CB compartments. (F and G), comparison of the NP, UB, and CB gene lengths (**F**) and % intronic length (**G**) (see Table S1D, columns M-Q). There were no significant differences between three types of biased genes for either lncRNAs or PCGs with respect to gene length and % intronic length. Error bars represent the interquartile range of the distribution from the median (horizontal midline). Black brackets compare lncRNAs to PCGs within the same fraction, and red brackets compare lncRNAs, or PCGs, between fractions. *= adjusted p-value < 0.05; ** = adjusted p-value < 0.0001.

Additional File Fig. S5. Expression, fold-change and transcript maturity in the chromatin bound versus chromatin bound non-polyA selected fractions. Log2 fold-change versus log10 FPKM of chromatin-bound (positive y-axis values) versus chromatin-bound non-polyA selected lncRNAs (negative y-axis values) (**A**) and PCGs (**B**), presented here as separate graphs (c.f., combined graph in Fig. 4B. Also see Table S2C, columns D and E. Strength of compartment bias data (**C**) and FPKM expression level (**D**) for lncRNAs and PCGs that show chromatin-bound (CB) and chromatin-bound non-polyA selected (CBnPAs) bias. (**E**) Transcript maturity data (IO/EC read density ratio, Table S1F) is graphed in the chromatin-bound and chromatin-bound non-polyA selected fractions for those lncRNAs and PCGs that are biased for the chromatin- bound (CB) or chromatin-bound non-polyA biased (CBnPAs) unbiased fraction (UB) or are unbiased (UB). Only multi-exonic genes were included in this analysis, and only differences between the CB and UB and UB and CBnPAs groups were evaluated for statistical significance in the PolyA selected fraction. Error bars represent the interquartile range of the

distribution from the median (horizontal midline). Black brackets compare lncRNAs to PCGs within the same fraction, and red brackets compare lncRNAs, or PCGs, between fractions (*= adjusted p-value < 0.05; ** = adjusted p-value < 0.0001).

Additional File Fig. S6. Quantification of lncRNA and PCG expression from smFiSH analysis. Quantification of the numbers of nuclei expressing lnc_7423 (A) and the number of lnc_7423 molecules per nucleus (B) in two individual female livers and two individual male livers. Mean percentage or counts are graphed based on five fields of view for each liver, and error bars represent the SEM. Quantification of the number of nuclei expressing lnc_14770 (C) and the number of lnc_14770 molecules per nucleus (D) in two female livers and two male livers. Mean percentage or counts are graphed for five fields of view for each liver, and error bars represent the SEM. Relative expression of Cyp2b10 (E) and lnc_5998 (F) in male or female liver, either untreated (UT) or from mice treated with TCPOBOP for 51 hr (TCPO). Mean counts are graphed for 5 fields of view for each liver, and error bars represent the SEM. Asterisks show the significance of student t-tests (p-values: * < 0.05, ** < 0.01, *** < 0.001, **** < 0.0001).

Fig. S1

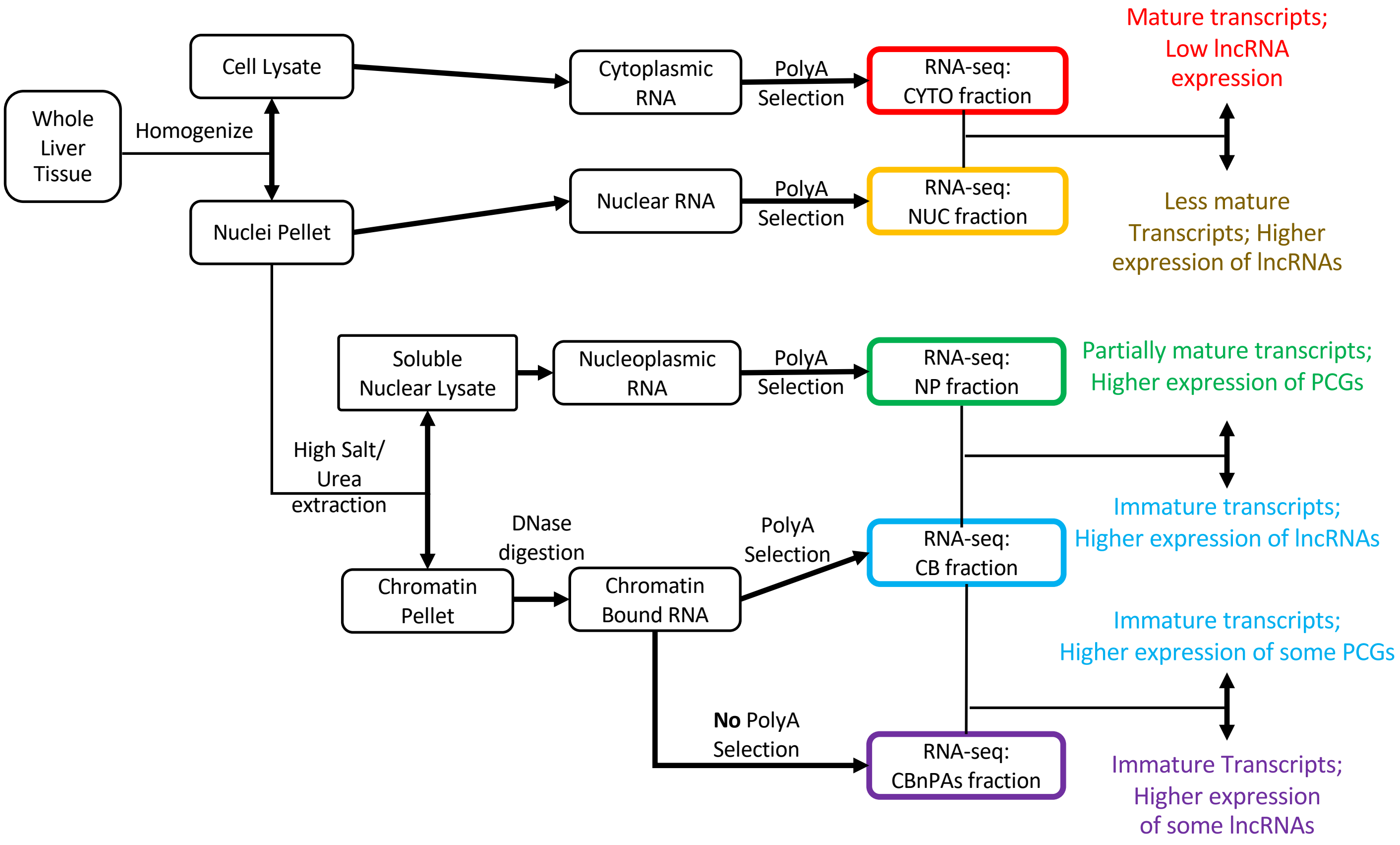

Whole Liver Tissue
Homogenize
Cell Lysate
Nuclei Pellet
Cytoplasmic RNA
Nuclear RNA
PolyA Selection
PolyA Selection
RNA-seq: CYTO fraction
RNA-seq: NUC fraction
Mature transcripts; Low lncRNA expression
Less mature Transcripts; Higher expression of lncRNAs
High Salt/ Urea extraction
Soluble Nuclear Lysate
Nucleoplasmic RNA
PolyA Selection
RNA-seq: NP fraction
Partially mature transcripts; Higher expression of PCGs
Immature transcripts; Higher expression of lncRNAs
Chromatin Pellet
DNase digestion
Chromatin Bound RNA
PolyA Selection
RNA-seq: CB fraction
Immature transcripts; Higher expression of some PCGs
No PolyA Selection
RNA-seq: CBnPAs fraction
Immature Transcripts; Higher expression of some lncRNAs

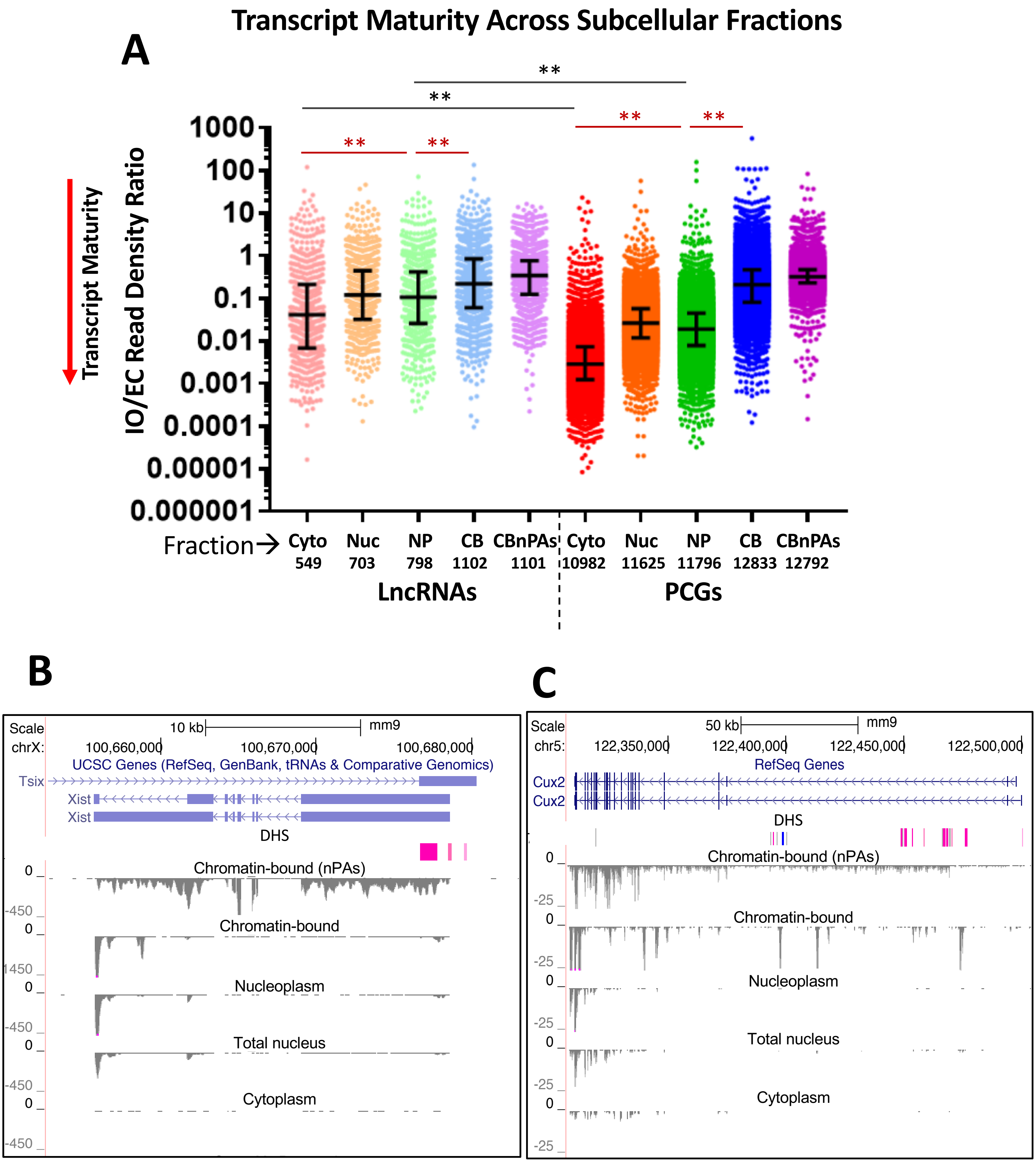
Transcript Maturity Across Subcellular Fractions
A
Transcript Maturity
IO/EC Read Density Ratio
1000
100
10
1
0.1
0.01
0.001
0.0001
0.00001
0.000001
**
Fraction
Cyto 549
Nuc 703
NP 798
CB 1102
CBnPAs 1101
Cyto 10982
Nuc 11625
NP 11796
CB 12833
CBnPAs 12792
LncRNAs
PCGs
B
Scale
10 kb
mm9
chrX:
100,660,000
100,670,000
100,680,000
UCSC Genes (RefSeq, GenBank, tRNAs & Comparative Genomics)
Tsix
Xist
Xist
DHS
Chromatin-bound (nPAs)
Chromatin-bound
Nucleoplasm
Total nucleus
Cytoplasm
0
-450
C
Scale
50 kb
mm9
chr5:
122,350,000
122,400,000
122,450,000
122,500,000
RefSeq Genes
Cux2
Cux2
DHS
Chromatin-bound (nPAs)
Chromatin-bound
Nucleoplasm
Total nucleus
Cytoplasm
0
-25

Fig. S3

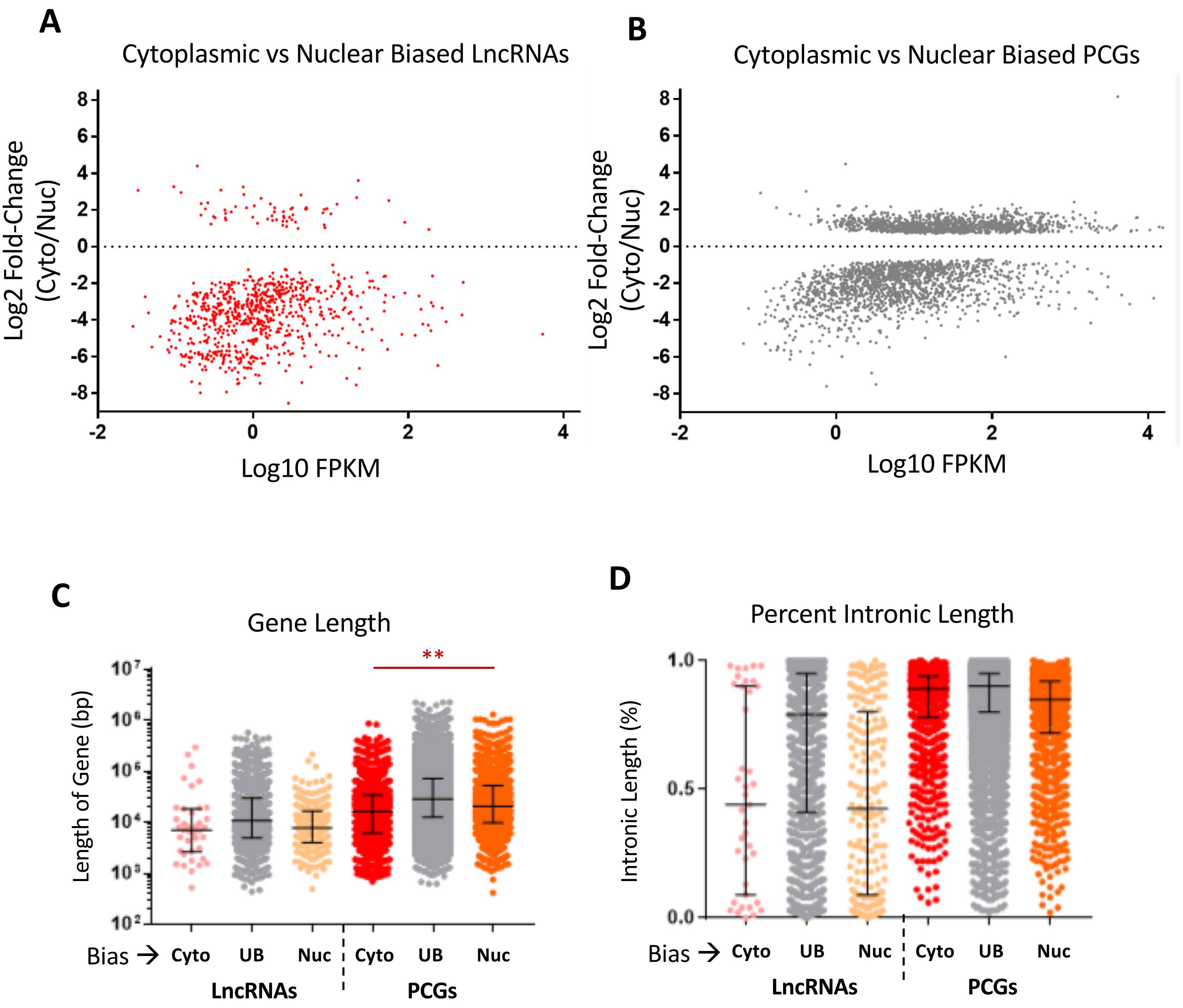

A
Cytoplasmic vs Nuclear Biased LncRNAs
Log2 Fold-Change (Cyto/Nuc)
Log10 FPKM
B
Cytoplasmic vs Nuclear Biased PCGs
Log2 Fold-Change (Cyto/Nuc)
Log10 FPKM
C
Gene Length
Length of Gene (bp)
**
Bias → Cyto UB Nuc Cyto UB Nuc
LncRNAs
PCGs
D
Percent Intronic Length
Intronic Length (%)
Bias → Cyto UB Nuc Cyto UB Nuc
LncRNAs
PCGs

Fig. S4A-S4D

A

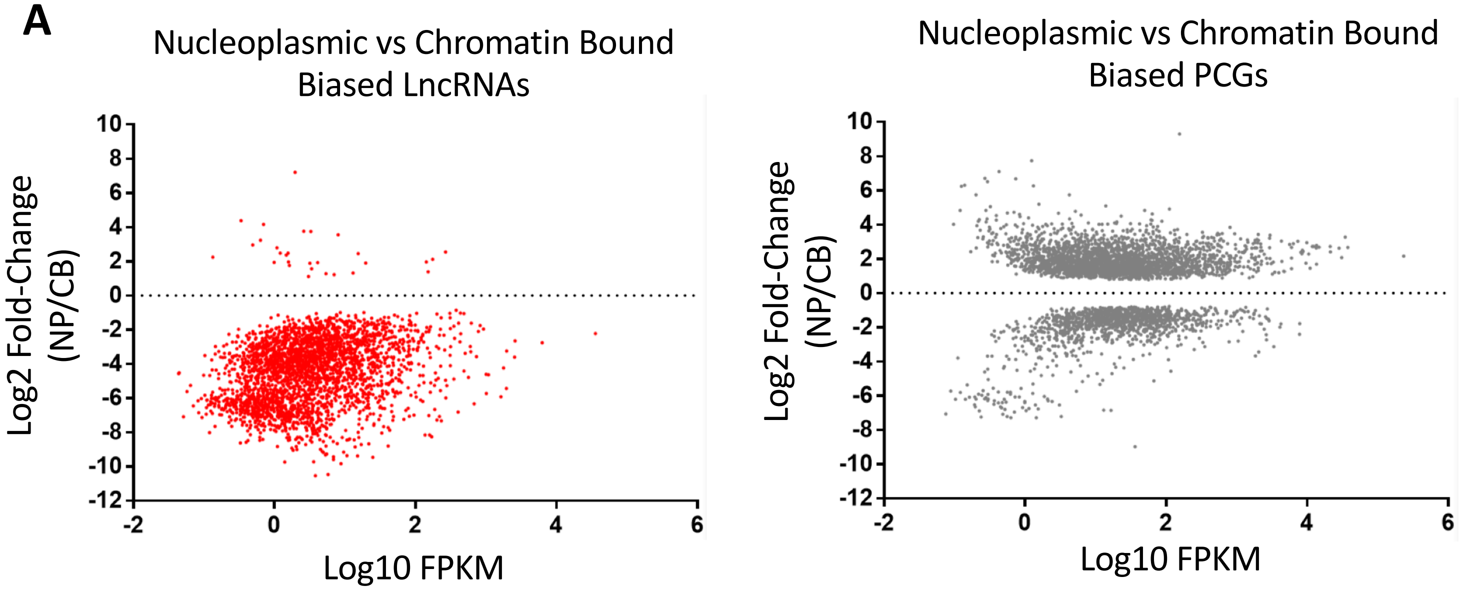

B

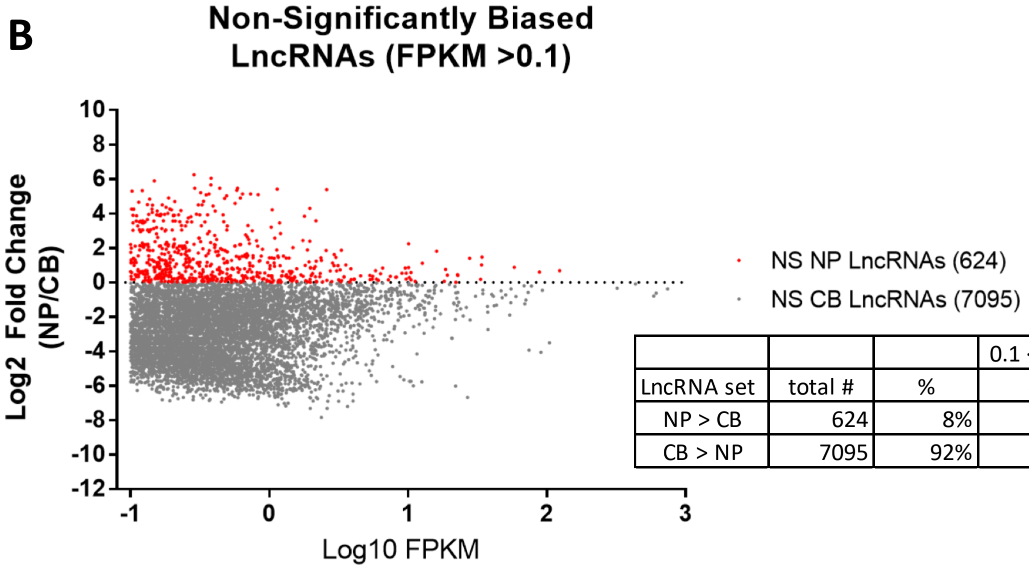

			0.1 < Max FPKM < 1		|FC| > 4	
LncRNA set	total #	%	#	% of total	#	% of total
NP > CB	624	8%	479	77%	453	73%
CB > NP	7095	92%	5695	80%	2304	32%

C

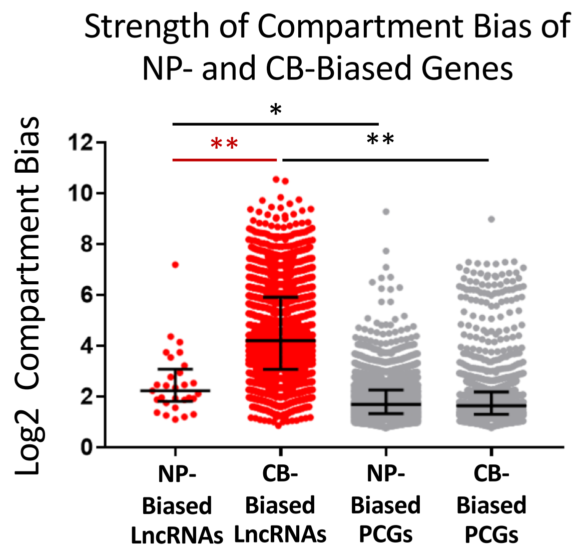

D

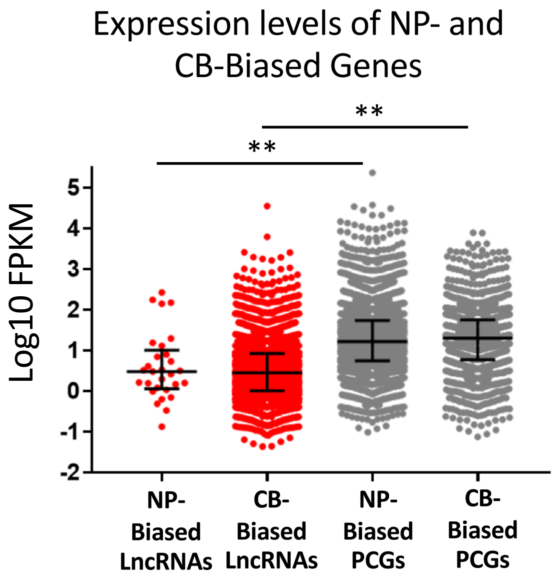

Fig. S4E-S4G

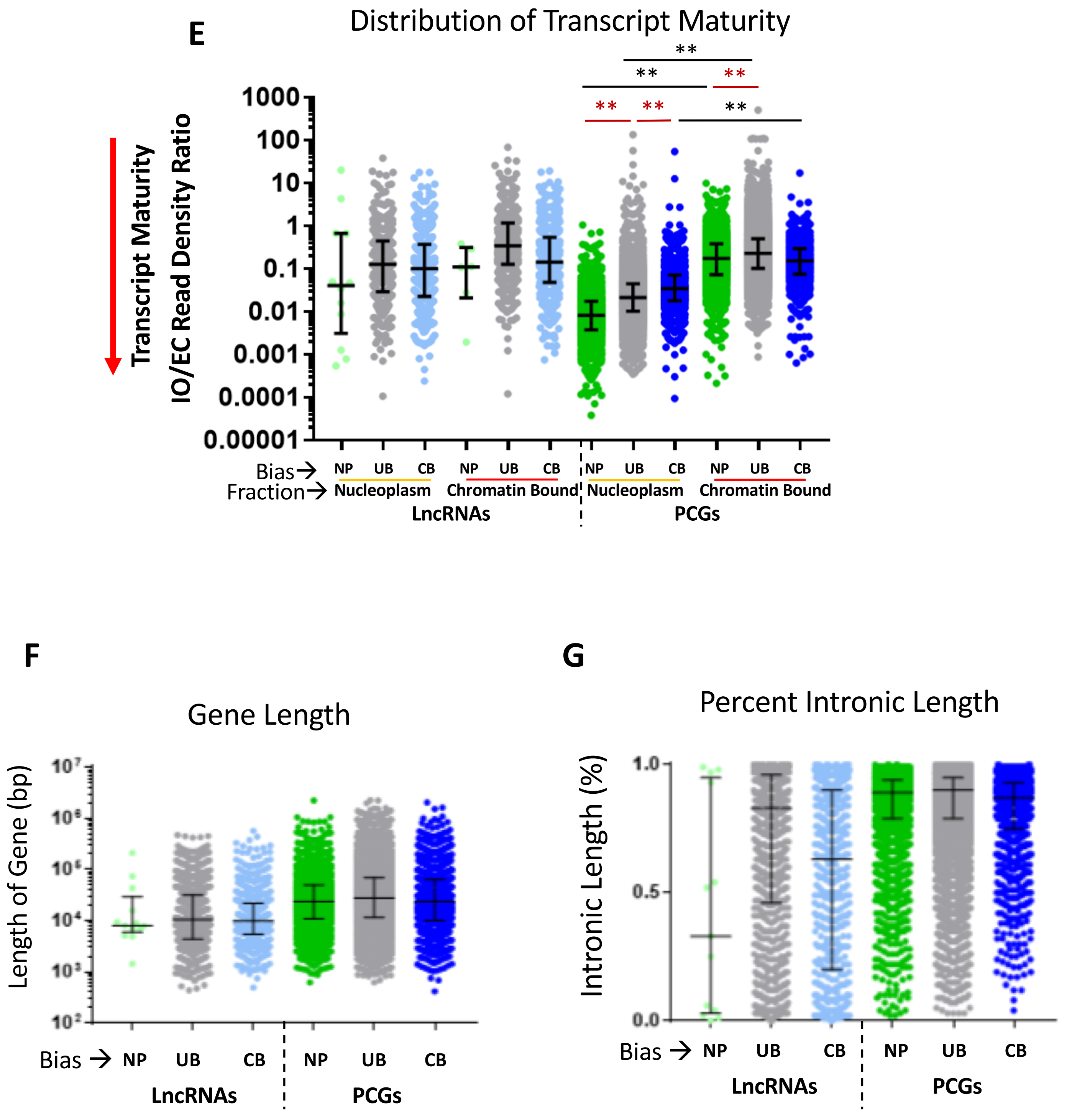

E
Distribution of Transcript Maturity
Transcript Maturity
IO/EC Read Density Ratio
1000
100
10
1
0.1
0.01
0.001
0.0001
0.00001
**
**
**
**
**
**
Bias→ NP UB CB NP UB CB NP UB CB NP UB CB
Fraction→ Nucleoplasm Chromatin Bound Nucleoplasm Chromatin Bound
LncRNAs
PCGs
F
Gene Length
Length of Gene (bp)
10^7
10^6
10^5
10^4
10^3
10^2
Bias → NP UB CB NP UB CB
LncRNAs
PCGs
G
Percent Intronic Length
Intronic Length (%)
1.0
0.5
0.0
Bias → NP UB CB NP UB CB
LncRNAs
PCGs

Fig. S5

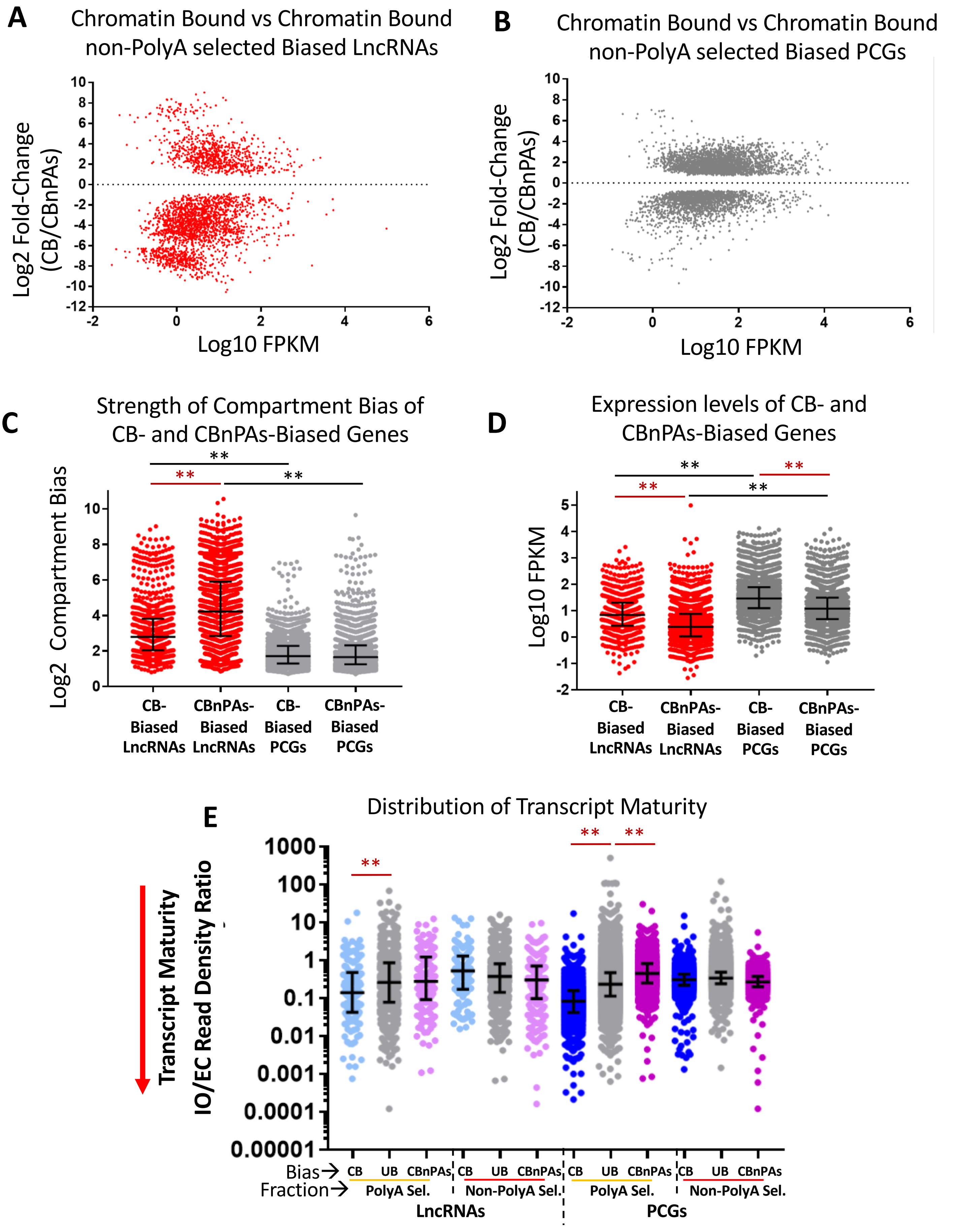
A
Chromatin Bound vs Chromatin Bound non-PolyA selected Biased LncRNAs
Log2 Fold-Change (CB/CBnPAs)
Log10 FPKM
B
Chromatin Bound vs Chromatin Bound non-PolyA selected Biased PCGs
Log2 Fold-Change (CB/CBnPAs)
Log10 FPKM
C
Strength of Compartment Bias of CB- and CBnPAs-Biased Genes
Log2 Compartment Bias
CB-Biased LncRNAs
CBnPAs-Biased LncRNAs
CB-Biased PCGs
CBnPAs-Biased PCGs
D
Expression levels of CB- and CBnPAs-Biased Genes
Log10 FPKM
CB-Biased LncRNAs
CBnPAs-Biased LncRNAs
CB-Biased PCGs
CBnPAs-Biased PCGs
E
Distribution of Transcript Maturity
Transcript Maturity
IO/EC Read Density Ratio
Bias
Fraction
CB UB CBnPAs
PolyA Sel.
Non-PolyA Sel.
LncRNAs
PCGs

Fig. S6

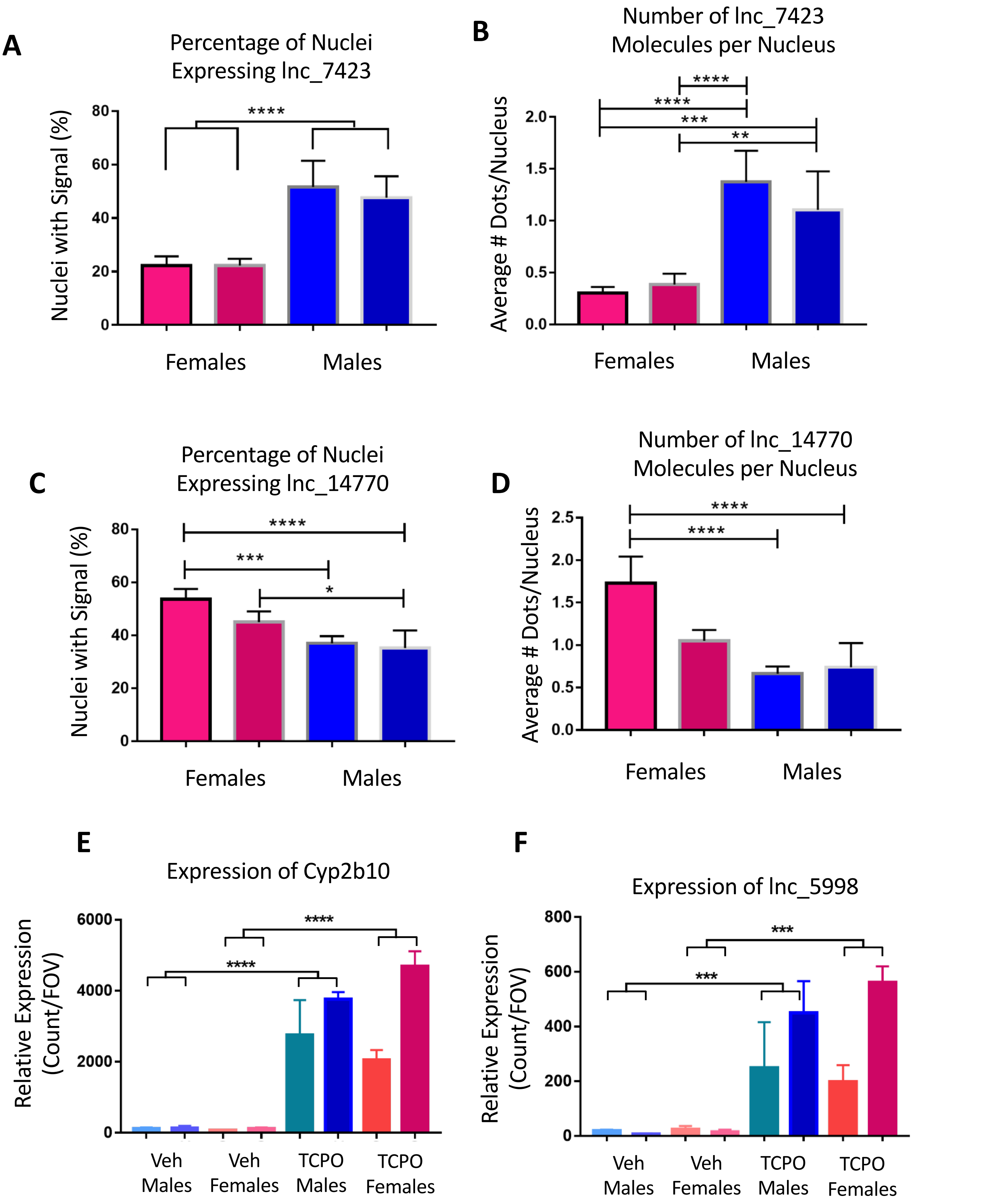
A
Percentage of Nuclei Expressing lnc_7423
Nuclei with Signal (%)
****
Females
Males
B
Number of lnc_7423 Molecules per Nucleus
Average # Dots/Nucleus
****
****
***
**
Females
Males
C
Percentage of Nuclei Expressing lnc_14770
Nuclei with Signal (%)
****
***
*
Females
Males
D
Number of lnc_14770 Molecules per Nucleus
Average # Dots/Nucleus
****
****
Females
Males
E
Expression of Cyp2b10
Relative Expression (Count/FOV)
****
****
Veh Males
Veh Females
TCPO Males
TCPO Females
F
Expression of lnc_5998
Relative Expression (Count/FOV)
***
***
Veh Males
Veh Females
TCPO Males
TCPO Females
